# Altered cardiac excitability and arrhythmia in models of *SCN1B*-linked developmental and epileptic encephalopathy

**DOI:** 10.1172/jci.insight.190918

**Published:** 2025-08-05

**Authors:** Roberto Ramos-Mondragon, Shuyun Wang, Nnamdi Edokobi, Qinghua Liu, Xiaotan Qiao, Maya Shih, Louis T. Dang, Yao-Chang Tsan, Katalin Štěrbová, Adam S. Helms, Sarah Weckhuysen, Luis F. Lopez-Santiago, Jack M. Parent, Lori L. Isom

**Affiliations:** 1 Department of Pharmacology,; 2 Department of Neurology,; 3 Department of Neuroscience,; 4 Department of Pediatrics,; 5 Department of Human Genetics, and; 6 Department of Internal Medicine, University of Michigan Medical School, Ann Arbor, Michigan, USA.; 7 Department of Pediatric Neurology, Charles University and Motol Hospital, Prague, Czech Republic.; 8 Applied & Translational Neurogenomics Group, VIB Center for Molecular Neurology, VIB, Antwerp, Belgium.; 9 Translational Neurosciences, Faculty of Medicine and Health Science, University of Antwerp, Antwerp, Belgium.; 10 Department of Neurology, Antwerp University Hospital, Antwerp, Belgium.; 11 Michigan Neuroscience Institute, University of Michigan Medical School, Ann Arbor, Michigan, USA.; 12 VA Ann Arbor Healthcare System, Ann Arbor, Michigan, USA.

**Keywords:** Cardiology, Stem cells, Arrhythmias, Epilepsy, Mouse models

## Abstract

Biallelic variants in *SCN1B*, which encodes the voltage-gated sodium channel β1/β1B subunits, are linked to DEE52, a developmental and epileptic encephalopathy with a high risk of sudden unexpected death in epilepsy (SUDEP). DEE52 patients present clinically with Dravet syndrome or the more severe early infantile DEE. *SCN1B* is expressed in brain and heart in humans and in mice. Thus, we have proposed that, in addition to generalized seizures, cardiac arrhythmia may play a role in SUDEP. Mice with homozygous expression of the DEE52 variant *Scn1b*-c.265C>T, predicting p.R89C, have spontaneous and hyperthermia-induced generalized seizures and SUDEP. Here we conducted cardiac characterization of *Scn1b*-c.265C>T mice and studied induced pluripotent stem cell cardiomyocytes (iPSC-CMs) derived from 2 *SCN1B*-c.265C>T DEE52 patients. *Scn1b^C89/C89^* mouse CMs showed increased transient outward potassium current (I_to_) density and heart sections revealed ventricular fibrosis. *Scn1b^C89/C89^* mice were susceptible to pacing-induced cardiac arrhythmias. Patient-derived iPSC-CMs with biallelic *SCN1B*-c.265C>T variant expression showed increased sodium current (I_Na_), late I_Na_, and I_to_ current densities. We conclude that, while mouse and human cardiac AP waveforms have critical differences, increased I_to_ is common to both models of DEE52. Overall, our data suggest that electrical and structural substrates may lead to arrhythmias and contribute to SUDEP in DEE52.

## Abstract 2

This new work showing altered excitability in patient-derived iPSC cardiac myocytes strengthens the hypothesis that cardiac arrhythmias may contribute to SUDEP mechanisms in developmental and epileptic encephalopathies linked to variants in sodium channel α and β subunit genes.

## Graphical abstract

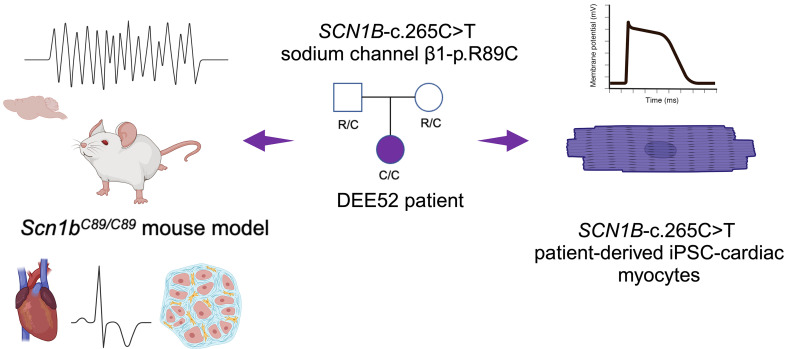

## Introduction

Sudden unexpected death in epilepsy (SUDEP) is the leading cause of death in people with uncontrolled seizures. While all patients with epilepsy are at risk for SUDEP, patients with developmental and epileptic encephalopathy (DEE) syndromes have the highest risk (1). DEEs are characterized by severe, pharmacoresistant seizures of multiple semiologies, developmental delay, intellectual disability, and often premature mortality (2). No biomarkers exist to predict the extent of SUDEP risk in individual patients other than the presence of variants in specific genes (3). To gain insight into the mechanism of SUDEP, we have focused on DEE syndromes with the highest SUDEP incidence. Patients with DEE52 have inherited, biallelic variants in *SCN1B*, encoding the voltage-gated sodium channel (VGSC) β1 and β1B subunits. DEE52 patients have clinical presentations comparable to Dravet syndrome (DS) or to the more severe early infantile DEE (4–7). Inherited monoallelic *SCN1B* variants are linked to genetic epilepsy with febrile seizures plus (GEFS+) (8–10) and cardiac disorders such as Brugada syndrome and atrial fibrillation (AF), which are also associated with sudden death (11). Although the mechanisms of SUDEP remain unclear, we hypothesize that, in addition to seizures, SUDEP in some instances involves cardiac arrhythmias (12). We proposed previously using multiple models, including *Scn1a*-haploinsufficient mice that model DS (13), *Scn1b*-null mice that model DS or DEE52 (14, 15), *Scn8a* mice that model DEE13 (16), and *SCN1A*-linked DS patient–derived induced pluripotent stem cell cardiomyocytes (iPSC-CMs) (17), that increased sodium current (I_Na_) density may be a biomarker for SUDEP risk by providing a substrate for cardiac arrhythmia. This work provided preclinical evidence that cardiac dysregulation, in addition to severe seizures, may play a role in the mechanism of SUDEP in DEEs caused by variants in VGSC genes. Here, we test the strength of our hypothesis using mouse and human models of a DEE52 variant that causes partial loss of function.

VGSCs are critical for the generation and propagation of neuronal and cardiac action potentials (APs). VGSCs are heterotrimeric protein complexes composed of a single pore-forming α subunit and 2 non–pore-forming β subunits that modulate the α subunit in a cell-type-specific manner (18). *Scn1b* deletion in mice results in severe seizures and death by the third week of life (19). Importantly, *Scn1b* deletion is also arrhythmogenic. *Scn1b*-null mice have ventricular (14, 20) and atrial arrhythmias (15).

VGSC β1 subunits are multifunctional. In addition to modulating VGSC expression and gating, β1 subunits modify potassium channels (21, 22). These β1 subunits are members of the immunoglobulin superfamily of cell adhesion molecules (Ig-CAMs) (23, 24). In the heart, the cell adhesive properties of β1 subunits are critical for formation of intercalated disks (25). As CAM substrates for regulated intramembrane proteolysis (26), β1 subunits function as transcriptional regulators of genes critical in the regulation of cardiac excitability, including those encoding potassium channels (27).

The biallelic DEE52 patient variant, *SCN1B*-c.265C>T, predicting p.R89C, was reported in 2 unrelated patient families (28, 29). In one nonconsanguineous family, one child was diagnosed with DS, while the other had a milder epilepsy phenotype (28). We then identified another biallelic *SCN1B*-c.265C>T patient with a clinically more severe phenotype than DS (29). We generated a transgenic mouse model and reported that *Scn1b^C89/C89^* mice, with homozygous expression of the variant, have spontaneous and hyperthermia-induced seizures and die prematurely (29). Heterologous expression of β1-p.R89C cDNA in HEK cells resulted in VGSC α subunit subtype–specific effects on I_Na_ density (29). Here, we characterized the cardiac phenotype of homozygous *Scn1b^C89/C89^*, heterozygous *Scn1b^R89/C89^*, and WT *Scn1b^R89/R89^* mice. To understand the translatability of this mouse model, we also analyzed induced pluripotent stem cell–derived (iPSC-derived) ventricular CMs from 2 biallelic *SCN1B*-c.265C>T DEE52 patients. Our combined results suggest that, while mouse and human cardiac AP waveforms are very different, increased outward potassium current (I_to_) density is common to both models of DEE52. Furthermore, our data further strengthen the hypothesis that cardiac arrhythmias may contribute to SUDEP mechanisms in DEEs linked to variants in VGSC α and β subunit genes.

## Results

### DEE52 mice have a cardiac phenotype.

*Scn1b* deletion leads to severe seizures, atrial and ventricular cardiac arrhythmias, and death by the third week of life in 100% of mice (14, 15, 19, 20). However, because DEE52 patients are not null for *SCN1B*, we generated mice expressing the variant *SCN1B*-c.265C>T, predicting p.R89C (29). We showed that 100% of *Scn1b^C89/C89^* mice exhibit spontaneous and hyperthermia-induced seizures and that 20% of these animals die by postnatal day 60 (P60) (29). Here, we investigated the cardiac physiology of *Scn1b*-c.265C>T monoallelic mice that model the asymptomatic parents (*Scn1b^R89/C89^*) and biallelic mice that model the proband (*Scn1b^C89/C89^*) compared to control mice (*Scn1b^R89/R89^*), with particular emphasis on identifying arrhythmogenic substrates.

**Figure 1:**
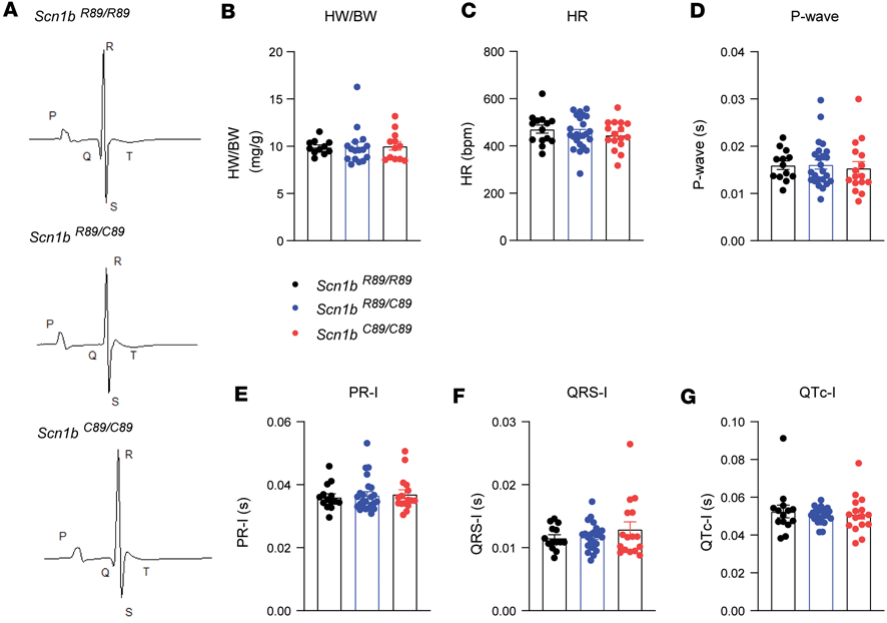
Heart weight to body weight ratio and ECG lead II properties in P18–P25 *Scn1b ^R89/R89^*, *Scn1B^R89/C89^*, and *Scn1B^C89/C89^* mice. (**A**) Representative ECG-lead II recordings. Black line represents the average ECG signal over 1 minute (green traces). (**B**) Heart weight to body weight ratio. (**C**) Heart rate. (**D**) P-wave duration. (**E**) PR interval. (**F**) QRS interval. (**G**) QT interval. Correction of the QT interval was performed using Mitchell’s formula. *n* = 15 for *Scn1b^R89/R89^* mice*, n* = 29 for *Scn1b ^R89/C89^* mice, and *n* = 17 for *Scn1b ^R89/89^* mice. Values represent mean ± SEM.

**Figure 2:**
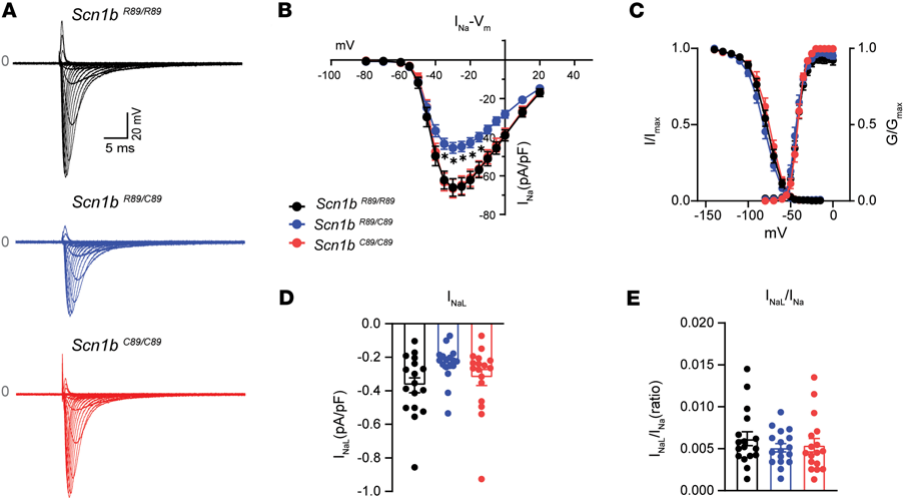
Sodium current (I_Na_) properties of acutely isolated Scn*1b^R89/R89^*, *Scn1b^R89/C89^*, and *Scn1b^C89/C89^* CMs from P18–P25 mice. (**A**) Representative recordings of I_Na_ in *Scn1b^R89/R89^*, *Scn1b^R89/C89^*, and *Scn1b^C89/C89^* CMs. *Scn1b^R89/C89^* CMs showed lower I_Na_ density compared with *Scn1b^R89/R89^ and Scn1b^C89/C89^*. (**B**) Voltage-current relationship of I_Na_. Low I_Na_ density was found at –35, –30, –25, –20, and 10 mV in the *Scn1b^R89/C89^* CMs. (**C**) Normalized activation and inactivation curves. No differences in voltage-dependent properties were identified. (**D**) Late sodium current density (I_NaL_) recorded at –20 mV was not different between genotypes. (**E**) Normalized I_NaL_ to I_Na_ ratio was not different between genotypes. Values represent mean ± SEM. *n* = 19 cells from 3 *Scn1b ^R89/R89^* mice, *n* = 24 cells from 3 *Scn1b ^R89/C89^* mice, and *n* = 18 cells from 3 *Scn1b ^C89/C89^* mice. *P < 0.05 compared with *Scn1b^R89/R89^* and *Scn1b^C89/C89^* using 1-way ANOVA with Tukey’s post hoc comparison test. Dots represent individual cells.

We recorded surface ECGs in anesthetized P18–P25 animals and assessed their physical characteristics (Figure 1). We found no differences in body weight (BW), heart weight (HW), HW/BW ratio, heart rate (HR), P-wave duration, and PR, QRS, and QT intervals between the 3 genotypes (Figure 1 and Supplemental Table 1; supplemental material available online with this article; https://doi.org/10.1172/jci.insight.190918DS1). Patch clamp recordings in acutely isolated ventricular CMs from P18–P25 mice showed reduced I_Na_ density in *Scn1b^R89/C89^* compared with *Scn1b^R89/R89^* or *Scn1b^C89/C89^* (Figure 2A), observed at voltages between –35 and –10 mV, as indicated in the I-V curves (–64.7 ± 4.4 pA/pF *Scn1b^R89/R89^* vs. –46.0 ± 2.7 pA/pF *Scn1b^R89/C89^* vs. –65.6 ± 4.2 pA/pF *Scn1b^C89/C89^* at –30 mV; *P* < 0.05; Figure 2B). We found no changes in the voltage dependence of activation between genotypes (Figure 2C and Supplemental Table 2). Finally, noninactivating, or late sodium current density (I_NaL_) density, as well as the I_NaL_/I_Na_ ratio were comparable between genotypes (Figure 2, D and E).

**Figure 3:**
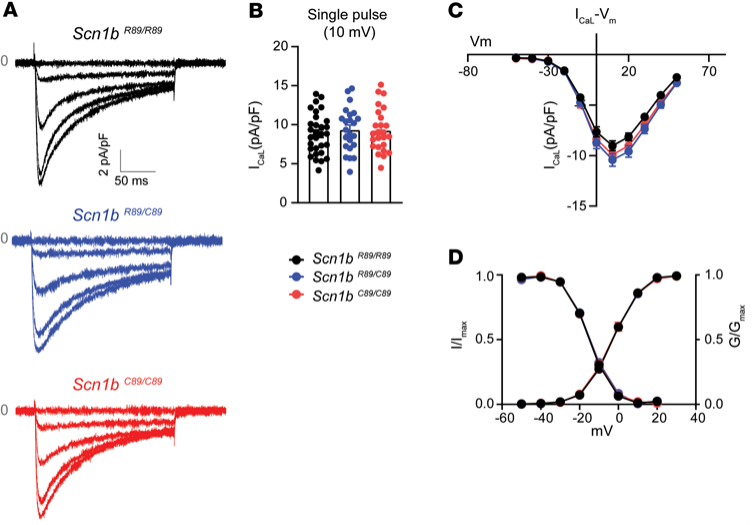
I_CaL_ properties of acutely isolated P18–P25 *Scn1b^R89/R89^*, *Scn1b^R89/C89^*, and *Scn1b^C89/C89^* mouse CMs. (**A**) Representative recordings of I_CaL_ in *Scn1b^R89/R89^*, *Scn1b^R89/C89^*, and *Scn1b^C89/C89^* mouse CMs. (**B**) I_CaL_ recorded at single pulse (SP) at 10 mV. This SP was applied before the application of current-voltage protocol. (**C**) Voltage-current relationship of I_CaL_. No differences in I_CaL_ density were noted between genotypes. (**D**) Voltage dependence of activation and inactivation for I_CaL_. Values represent mean ± SEM. *n* = 29 cells from 5 *Scn1b ^R89/R89^* mice, *n* = 22 cells from 4 *Scn1b ^R89/C89^* mice, and *n* = 23 cells from 4 *Scn1b ^C89/C89^* mice.

We recorded L-type calcium (I_CaL_) currents in ventricular CMs from P18–P25 mice by applying a single pulse to +10 mV, followed by subsequent pulses to various membrane potentials. We found no differences in I_CaL_​ density across the 3 genotypes (Figure 3, A–C). Similarly, no changes were observed in the voltage-dependent I_CaL_ activation or inactivation (Figure 3D and Supplemental Table 3).

**Figure 4:**
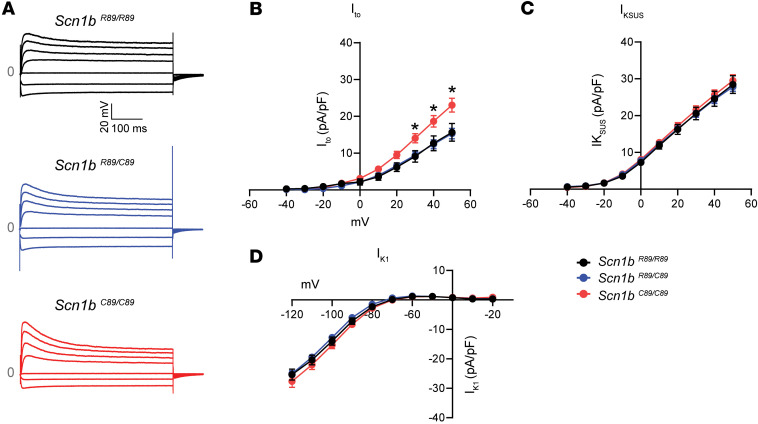
Recordings of I_to_, I_KSUS_, and I_K1_ in acutely isolated P18–P25 mouse CMs. (**A**) Representative I_K_ recordings from *Scn1b^R89/R89^*, *Scn1b^R89/C89^*, and *Scn1b^C89/C89^* mouse CMs. *Scn1b^C89/C89^* showed increased I_to_. (**B**) I-V relationship for I_to_. Increased I_to_ was observed at membrane potentials of 30, 40, and 50 mV. (**C**) I-V relationship for I_KSUS_. (**D**) I-V relationship for I_K1_. No significant differences in I_KSUS_ and I_K1_ were identified among the 3 groups of CMs. Values represent mean ± SEM. I_to_ and I_KSUS:_ *n* = 24 cells from 4 *Scn1b ^R89/R89^* mice, *n* = 44 cells from 5 *Scn1b ^R89/C89^* mice, and *n* = 42 cells from 5 *Scn1b ^C89/C89^* mice. I_K1_: *n* = 24 cells from 5 *Scn1b ^R89/R89^* mice, *n* = 29 cells from 6 *Scn1b ^R89/C89^* mice, and *n* = 18 cells from 5 *Scn1b ^C89/C89^* mice. *P < 0.05 *Scn1b^C89/C89^* against *Scn1b^R89/R89^* and *Scn1b^R89/C89^* using 1-way ANOVA with Tukey’s post hoc comparison test.

We next recorded potassium currents (I_K_) in ventricular CMs from P18–P25 mice. Representative recordings of inward and outward I_K_ from the 3 CM genotypes are shown in Figure 4A. Current analysis showed that *Scn1b^C89/C89^* CMs had increased transient outward I_K_ (I_to_) compared with *Scn1b^R89/R89^* and *Scn1b^R89/C89^* CMs (22.0 ± 2.1 pA/pF *Scn1b*^C89/C89^ vs. 13.9 ± 1.9 pA/pF *Scn1b^R89/R89^* and 15.6 ± 1.3 pA/pF *Scn1b^R89/C89^* at 50 mV; *P* < 0.05; Figure 4, A and B). There were no detectable differences in the outward sustained I_K_ (I_KSUS_) or inward rectifier I_K_ (I_K1_) across the 3 genotypes (Figure 4, C and D).

**Figure 5:**
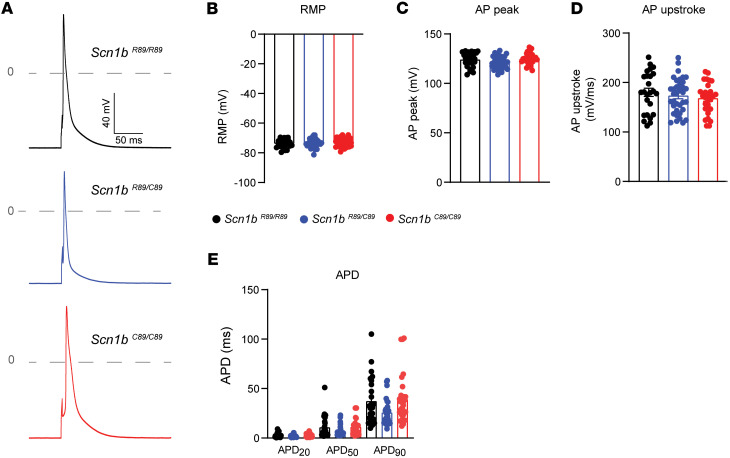
AP properties of acutely isolated P18–P25 *Scn1b^R89/R89^*, *Scn1b^R89/C89^*, and *Scn1b^C89/C89^* mouse CMs. (**A**) Representative recordings of APs in *Scn1b^R89/R89^*, *Scn1b^R89/C89^*, and *Scn1b^C89/C89^* mouse CMs. APs were recorded at 1.0 Hz. (**B**) Resting membrane potential. (**C**) AP peak. (**D**) AP upstroke. (**E**) Action potential duration at 20% (APD_20_), 50% (APD_50_), and 90% (APD_90_) of membrane repolarization. No significant differences in AP properties were identified between *Scn1b^R89/R89^*, *Scn1b^R89/C89^*, and *Scn1b^C89/C89^* CMs. Values represent mean ± SEM. *n* = 25 cells from 4 *Scn1b ^R89/R89^* mice, *n* = 36 cells from 5 *Scn1b ^R89/C89^* mice, and *n* = 30 cells from 5 *Scn1b ^C89/C89^* mice. Each dot represents an individual cell. Significant differences were assessed by a 1-way ANOVA with Tukey’s post hoc comparison test.

AP recordings from acutely isolated *Scn1b^R89/R89^*, *Scn1b^R89/C89^*, and *Scn1b^C89/C89^* P18–P25 ventricular CMs are shown in Figure 5A. We found no significant genotypic differences in resting membrane potential (RMP), AP peak amplitude, AP upstroke, or AP duration (APD) (Figure 5, B–E). High variability in the AP parameters, particularly in AP upstroke and APD, were noted for all 3 genotypes.

### DEE52 mice have increased levels of ventricular fibrosis.

**Figure 6:**
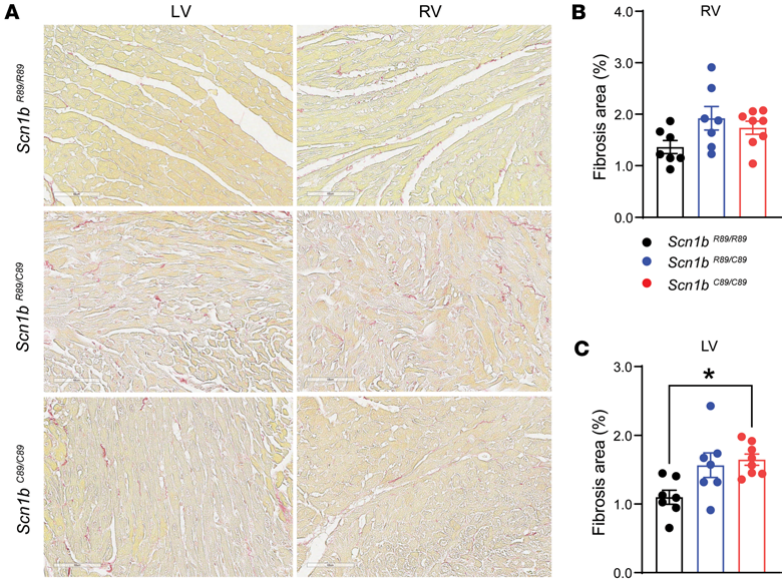
Fibrosis in P18–P25 *Scn1b^R89/R89^*, *Scn1b^R89/C89^*, and *Scn1b^C89/C89^* mouse hearts. (**A**) Representative images of Picrosirius red staining of histological coronal sections of left and right ventricular (LV and RV) tissue from *Scn1b^R89/R89^*, *Scn1b^R89/C89^*, and *Scn1b^C89/C89^* mice. Scale bars: 60 μm. (**B**) Quantification of fibrosis expressed as percentage area in the RV. (**C**) Quantification of fibrosis expressed as percentage area in the LV. Values represent mean ± SEM. Each dot represents 1 heart. *P < 0.05 by 1-way ANOVA with Tukey’s post hoc comparison test.

In our previous work, we showed that *Scn1b*-null neonatal mice have heightened levels of cardiac fibrosis compared with WT littermates (15). To determine whether expression of the *Scn1b*-c.265C>T variant in vivo resulted in similar changes, we collected P18–P25 mouse hearts for fibrosis staining. Figure 6A shows representative images of Picrosirius red–stained tissue from left and right ventricular chambers from *Scn1b^R89/R89^*, *Scn1b^R89C89^*, and *Scn1b^C89/C89^* hearts. Right ventricular staining was comparable among the 3 genotypes (Figure 6B). In contrast, Picrosirius red staining in *Scn1b^C89/C89^* left ventricles was significantly increased compared with *Scn1b^R89/R89^*, suggesting increased fibrosis (Figure 6C).

### RT-qPCR from transgenic mouse hearts.

We performed reverse transcription quantitative polymerase chain reaction (RT-qPCR) using ventricular tissue from P18–P25 mice to ask whether expression of the *Scn1b*-c.265C>T variant resulted in altered expression of other cardiac genes. Our results show that mRNA abundance of *Scn5a*, encoding the predominant cardiac VGSC, Nav1.5, was unaltered between genotypes (Supplemental Figure 1A). *Scn1b^C89/C89^* samples showed increased mRNA abundance of *Kcnd2*, encoding K_v_4.2 (Supplemental Figure 1B), which may underlie the observed increases in I_to_. *Scn1b^R89/C89^* ventricular tissue showed increased mRNA abundance of *Cacnac1*, encoding the L-type Ca^2+^ channel, Ca_v_1.2 (Supplemental Figure 1F). Finally, no differences were observed between genotypes for mRNA abundance of fibrotic markers (Supplemental Figure 1, G–J).

### DEE52 mice have increased propensity to cardiac arrhythmias.

**Figure 7:**
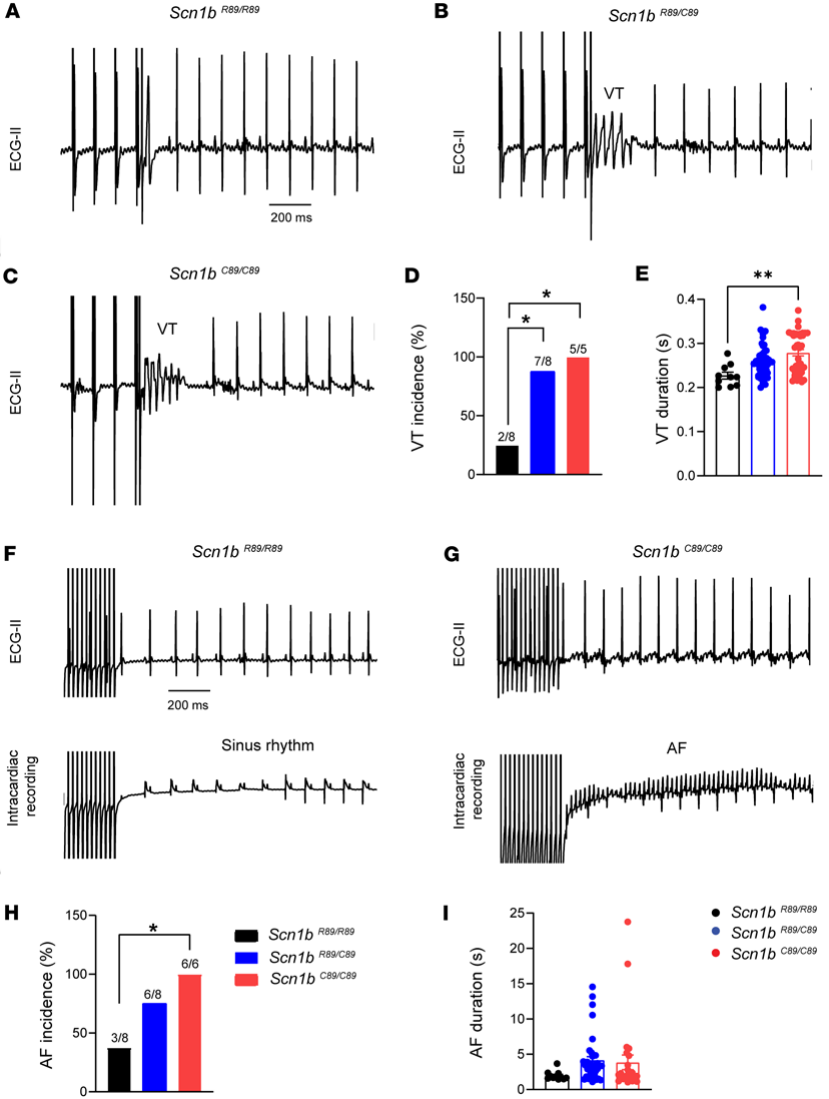
Electrophysiological assessment of cardiac arrhythmias in P18–P25 *Scn1b^R89/R89^*, *Scn1b^R89/C89^*, and *Scn1b^C89/C89^* mice. (**A**–**C**) Representative ECG lead II recordings showing VT in *Scn1b^R89/C89^* and *Scn1b^C89/C89^* mice following extra stimulation. (**D**) VT incidence. Both *Scn1b^R89/C89^* and *Scn1b^C89/C89^* mice showed higher incidence of VT compared with *Scn1b^R89/R89^* mice. (**E**) VT duration. *Scn1b^C89/C89^* mice showed longer VT episodes than *Scn1b^R89/R89^* mice. VT was defined as either nonsustained VT (≥3 consecutive ventricular depolarizations with abnormal QRS morphology) or ventricular fibrillation (VF; rapid, disorganized activity lacking discernible QRS complexes and lasting ≥1 second). (**F** and **G**) Representative ECG lead II and intracardiac signal in *Scn1b^R89/R89^* and *Scn1b^C89/C89^* mice. Atrial rapid pacing induced AF in the *Scn1b^C89/C89^* mouse but not in the *Scn1b^R89/R89^* mouse. (**H**) AF incidence. AF was defined as a rapid, irregular atrial rhythm lacking consistent P waves and displaying disorganized electrogram morphology lasting ≥1 second. All *Scn1b^C89/C89^* mice tested were inducible to AF. (**I**) AF duration. No significant differences were observed in the duration of induced AF episodes. Values represent mean ± SEM for arrhythmia duration. *P < 0.05 using Fisher’s exact test for comparisons of arrhythmia incidence among groups and **P < 0.01 using 1-way ANOVA with Tukey’s post hoc test for comparisons of arrhythmia duration.

To understand the physiological implications of our cellular electrophysiological findings, we performed intracardiac recordings in P18–P25 *Scn1b^R89/R89^*, *Scn1b^R89/C89^*, and *Scn1b^C89/C89^* mice to assess cardiac arrhythmias. We observed stimulation-induced ventricular tachycardia (VT) in 7 of 8 *Scn1b^R89/C89^* mice (*P* < 0.05) and in 5 of 5 *Scn1b^C89/C89^* mice tested (*P* < 0.05), compared with 2 of 8 *Scn1b^R89/R89^* mice (Figure 7, A–D). Examples of VT in *Scn1b^R89/C89^* and *Scn1b^C89/C89^* mice are shown in Figure 7, B and C compared with no VT in *Scn1b^R89/R89^* mice (Figure 7A). The duration of VT episodes in *Scn1b^C89/C89^* mice (0.28 ± 0.0 seconds; *P* < 0.05) was significantly longer than in *Scn1b^R89/R89^* mice (0.23 ± 0.0 seconds) (Figure 7E and Supplemental Table 4). The ventricular effective refractory period at a cycle length of 80 ms (VERP_80_) was shorter in *Scn1b^C89/C89^* (18.0 ± 1.5 ms) mice compared with *Scn1b^R89/R89^* mice (21.5 ± 1.0 seconds). No differences in sinus node recovery time (SNRT) were observed between genotypes (Supplemental Table 4).

We also assessed the incidence of AF in response to rapid atrial stimulation. AF was induced in 6 of 6 *Scn1b^C89/C89^* mice tested, compared with 3 out of 8 *Scn1b^R89/R89^* mice (*P* = 0.03; Figure 7, F–H). There were no significant differences in the duration of AF between genotypes (Figure 7I and Supplemental Table 4). These results demonstrate that the *Scn1b^C89/C89^* genotype is associated with a heightened susceptibility to ventricular and atrial arrhythmias in mice in vivo.

### DEE52 patient–derived iPSC-CMs have increased I_Na_ and I_NaL_ densities.

To determine the translatability of the *Scn1b*-c.265C>T mouse model to human physiology, we generated iPSCs from 2 unrelated bialleic *SCN1B*-c.265C>T DEE52 patients (*SCN1B^C89/C89^* Pt. 1 and *SCN1B^C89/C89^* Pt. 2) as well as 2 nonepileptic *SCN1B^R89/R89^* controls and the mother of *SCN1B^C89/C89^* Pt. 2, who is monoallelic for the variant and reported to be seizure free (*SCN1B^R89/C89^* Supplemental Table 5). The *SCN1B^C89/C89^* Pt. 1 and the 2 *SCN1B^R89/R89^* control lines were generated from skin fibroblasts. The *Scn1b^C89/C89^* *SCN1B^C89/C89^* Pt. 2 and *SCN1B^R89/C89^* lines were derived from peripheral blood mononuclear cells (PBMCs). All iPSC lines were differentiated into ventricular CMs using the small molecule patterning method based on Wnt signaling pathway modulation (30).

**Figure 8:**
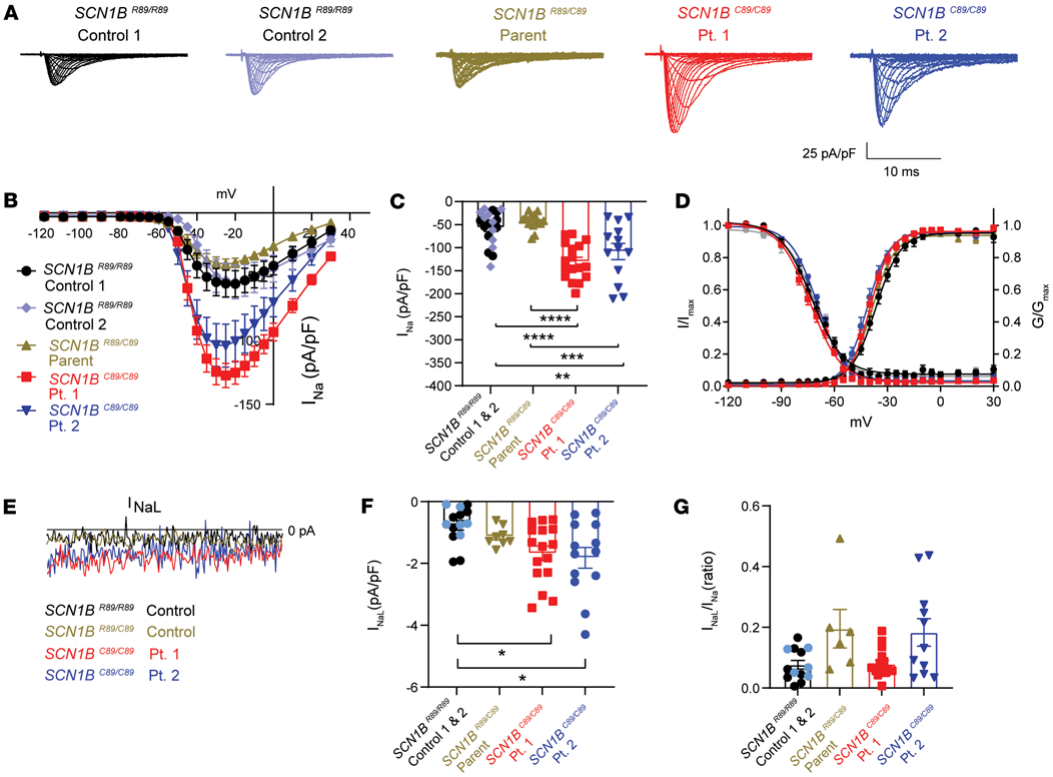
Transient and persistent I_Na_ are increased in patient iPSC-CMs. (**A**) Representative I_Na_ density traces of *SCN1B^R89/R89^* controls 1 and 2, *SCN1B^R89/C89^* control, *SCN1B^C89/C89^* Patient 1, and *SCN1B^C89/C89^* Patient 2. (**B**) I_Na_ current-voltage relationship for control and patient iPSC-CM lines. (**C**) Transient peak I_Na_ is increased 2-fold in patient 1 and patient 2 vs. control iPSC-CMs. (**D**) Voltage-dependent activation and inactivation properties. (**E**) Zoomed traces of I_NaL_ showing the current from 50 to 60 ms following the depolarizing pulse. (**F**) The mean I_NaL_ is significantly increased in the patient iPSC-CMs. (**G**) I_NaL_ normalized to the peak current. Data in **C**, **E**, and **F** are presented as mean ± SEM. *n* = 10 cells from *SCN1B^R89/R89^* control 1, *n* = 10 cells from *SCN1B^R89/R89^* control 2, *n* = 15 cells from *SCN1B^R89/C89^* Parent, *n* = 17 cells from *SCN1B^C89/C89^* Patient 1, and *n* = 13 cells from *SCN1B^C89/C89^* Patient 2. All cells were derived from at least 3 independent hiPSC differentiation batches. *P < 0.05; **P < 0.005; ***P < 0.0005; ****P < 0.0001 using a 1-way ANOVA with Tukey’s post hoc comparison test. Dots represent individual cells.

We performed whole-cell voltage-clamp recordings to investigate whether DEE52 patient iPSC-CMs had altered I_Na_ or I_NaL_ density. Initially, we used an external recoding solution containing 120 mM NaCl for this experiment. We found I_Na_ density from *SCN1B^C89/C89^* Pt. 1 CMs to be so large that proper voltage control could not be consistently maintained, and voltage-dependent properties could not be accurately measured (Supplemental Figure 2). We then changed the protocol to include an external solution containing 60 mM NaCl for all subsequent iPSC-CM voltage clamp experiments, which allowed us to reliably clamp the cells. Using this lower [NaCl] external solution, we found transient I_Na_ density to be significantly increased in *SCN1B^C89/C89^* Pt. 1 and *SCN1B^C89/C89^* Pt. 2 iPSC-CMs (–127.6 ± 9.6 pA/pF and –104.2 ± 17.0 pA/pF, respectively) compared with *SCN1B^R89/C89^* (–40.3 ± 4.1 pA/pF) and *SCN1B^R89/R89^* iPSC-CMs (–54.1 ± 8.3 pA/pF) (Figure 8, A–C). We found no differences in cell capacitance, slope factor, or V_½_ values for the voltage dependence of activation or steady-state inactivation in any line (Figure 8D and Supplemental Table 6). Peak sodium conductance (G_max_) was significantly increased in *SCN1B^C89/C89^* Pt. 1 and *SCN1B^C89/C89^* Pt. 2 iPSC-CMs compared with *SCN1B^R89/C89^* and *SCN1B^R89/R89^* cells (*P* < 0.05), consistent with increased I_Na_ density (Supplemental Table 6). Finally, we found a significant increase in the mean I_NaL_ density at –50 mV in *SCN1B^C89/C89^* Pt. 1 (–1.68 ± 0.24 pA/pF) and *SCN1B^C89/C89^* Pt. 2 (–1.82 ± 0.33 pA/pF) iPSC-CMs over control (–0.77 ± 0.16 pA/pF) iPSC-CMs (Figure 8, E and F), with no change in the ratio of late to transient peak I_Na_ densities for any genotype (Figure 8G).

Because functional properties of iPSC-CM clones generated from the same patient can vary, we evaluated clones separately to test the reproducibility of our data. Supplemental Figure 3 shows separate versus pooled data from *SCN1B^R89/R89^* control and *SCN1B^C89/C89^* Pt. 1 iPSC-CM clones. We observed no significant differences between *SCN1B^R89/R89^* control 1 (–59.41 ± 11.0 pA/pF) and *SCN1B^R89/R89^* control 2 (–43.0 ± 10.10 pA/pF) transient peak I_Na_ density values (Supplemental Figure 3, B and C). There were no significant differences in transient peak I_Na_ density between the 2 *SCN1B^C89/C89^* Pt. 1 clones (Supplemental Figure 3, B and C; Pt. 1: –134.8 ± 13.1 pA/pF vs. Pt. 2: –120.1 ± 13.0 pA/pF). Taken together, these results allowed us to pool results from the *SCN1B^C89/C89^* Pt. 1 clones and *SCN1B^R89/R89^* control clones, respectively.

### Increased I_to_ density and reduced I_CaL_ density in SCN1B-c.265C>T patient IPSC-CMs.

**Figure 9:**
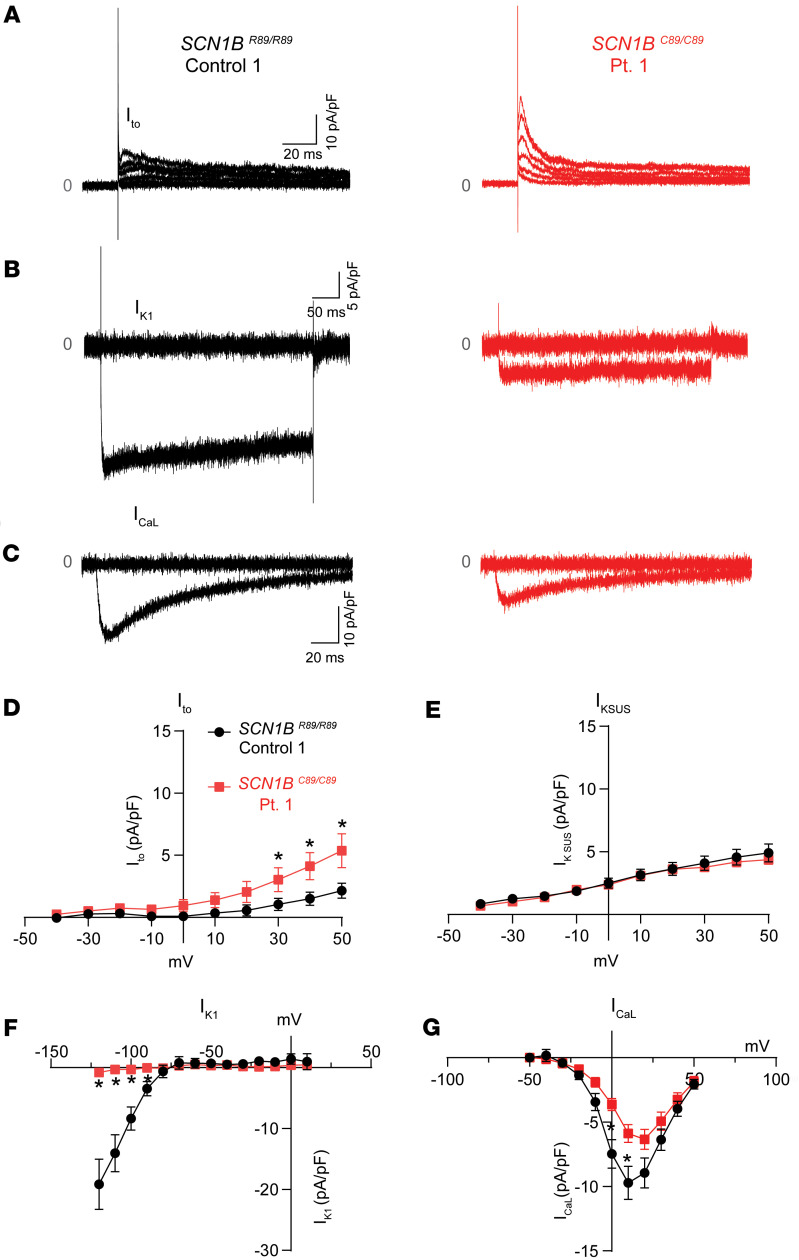
I_K_ and I_CaL_ properties of control and Pt. 1 iPSC-CMs. (**A**) Representative recordings of I_to_ in *SCN1B^R89/R89^* control and *SCN1B^C89/C89^* Pt. 1 iPSC-CMs. Pt. 1 iPSC-CMs showed higher I_to_ density. (**B**) Representative recordings of I_K1_ in *SCN1B^R89/R89^* control and and *SCN1B^C89/C89^* Pt.1 iPSC-CMs. Only currents obtained at –120 mV and –70 mV for I_K1_, are shown. Pt. 1 iPSC-CMs showed lower I_K1_ density. (**C**) Representative recordings for I_CaL_ in *SCN1B^R89/R89^* control and *SCN1B^C89/C89^* Pt. 1 iPSC-CMs. Only I_CaL_ obtained at –50 and 10 mV are shown. Pt. 1 iPSC-CMs showed lower I_CaL_ density. (**D**) Current-voltage relationships for I_to_. Higher I_to_ values were found in Pt. 1 iPSC-CMs than control iPSC-CMs at 30, 40, and 50 mV. (**E**) Current-voltage relationships for I_KSUS_. No significant changes in I_KSUS_ were found between genotypes. (**F**) Current-voltage relationship for I_K1_. Significantly smaller I_K1_ in Pt. 1 iPSC-CMs was observed from –120 mV to –90 mV (**G**) Current-voltage relationship for I_CaL_. Significantly smaller I_CaL_ in Pt. 1 iPSC-CMs was observed at 0 mV and 10 mV. Values represent mean ± SEM. I_to_ and I_KSUS_: *n* = 15 cells from *SCN1B^R89/R89^* control and *n* = 15 cells from *SCN1B^C89/C89^* Pt. 1. I_K1_: *n* = 8 cells from *SCN1B^R89/R89^* control and *n* = 6 cells from *SCN1B^C89/C89^* Pt. 1. I_CaL_: *n* = 16 cells from *SCN1B^R89/R89^* control and *n* = 16 cells from *SCN1B^C89/C89^* Pt. 1. All cells were derived from at least 3 independent hiPSC differentiation batches. *P < 0.05 using a 1-way ANOVA with Tukey’s post hoc comparison test.

We recorded I_K_ and I_CaL_ from *SCN1B^C89/C89^* Pt. 1 and *SCN1B^R89/R89^* control iPSC-CMs that had been prepared for AP measurement using the micron-scale 2-dimensional cardiac muscle bundle method but plated at lower density to facilitate voltage clamp recording. *SCN1B^C89/C89^* Pt. 1 iPSC-CMs showed increased I_to_ (4.0 ±0.7 pA/pF *SCN1B^R89/R89^* control vs. 7.8 ± 1.8 pA/pF *SCN1B^C89/C89^* Pt.1 at 70 mV; *P* < 0.05; Figure 9, A and D) and decreased I_K1_ (–19.1 ± 4.1 pA/pF *SCN1B^R89/R89^* control vs. –0.81 ± 0.7 pA/pF *SCN1B^C89/C89^* Pt. 1 at –120 mV; *P* < 0.05; Figure 9, B and F). I_KSUS_ densities were similar between genotypes (Figure 9E). I_CaL_ density was reduced in *SCN1B^C89/C89^* Pt. 1 iPSC-CMs compared with *SCN1B^R89/R89^* control (9.7 ± 1.2 pA/pF *SCN1B^R89/R89^* control vs. 5.9 ± 0.7 pA/pF *SCN1B^C89/C89^* Pt. 1 at 10 mV; *P* < 0.05; Figure 9, C and G).

### RT-qPCR analysis of mRNA abundance in SCN1B-c.265C>T patient iPSC-CMs.

We tested patient*,* parent, and control iPSC-CMs for changes in mRNA abundance of genes encoding VGSC α and β subunits but found no significant differences between genotypes (Supplemental Figure 4, A–H). These data suggest that the changes in I_Na_ density recorded in the patient lines are not due to transcriptional changes in VGSC gene expression but instead may occur via other pathways, including protein trafficking and posttranslational modifications. In contrast, we did observe differences in mRNA abundance of *KCND2* (decreased) and *KCND3* (increased) underlying I_to_, although in opposite directions (Supplemental Figure 5, A and B). Finally, no differences were observed in mRNA abundance of *KCNJ2,* underlying I_K1_, or *CACNA1C,* underlying I_CaL_ (Supplemental Figure 5, C and D).

### AP shortening in SCN1B-c.265C>T patient iPSC-CMs.

**Figure 10:**
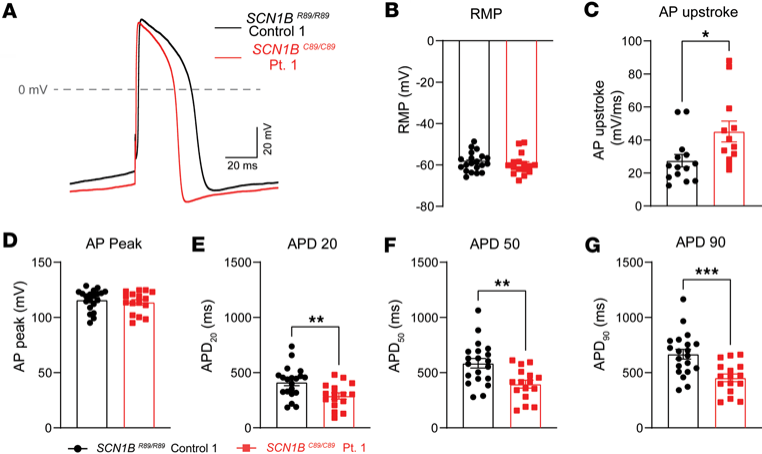
Patient iPSC-CMs show APD shortening. APs were evoked by pulses of 1.5 times the stimulus threshold at 1 Hz in current clamp mode. (**A**) Representative AP traces from *SCN1B^R89/R89^* control and *SCN1B^C89/C89^* Pt. 1 iPSC-CMs. (**B**) Resting membrane potential (RMP). (**C**) Maximal AP depolarization velocity. AP upstroke is increased in Pt. 1 iPSC-CMs. (**D**) Peak AP amplitude. (**E**–**G**) Action potential duration (APD) at 20% (APD_20_), 50% (APD_50_), and 90% (APD_90_) of membrane repolarization. Pt. 1 iPSC-CMs showed significant shortening of the APD at all percentages of membrane repolarization. Values represent mean ± SEM. *n* = 19 cells from *SCN1B^R89/R89^* control 1 and *n* = 16 cells from *SCN1B^C89/C89^* Pt. 1. All cells were derived from at least 3 independent hiPSC differentiation batches. *P < 0.05 by using a 2-tailed Student’s *t* test. Dots represent individual cells.

We generated iPSC cardiac tissues using the micron-scale 2-dimensional cardiac muscle bundle method, which has been shown to control for cell shape and enable reproducible characterization of cellular excitability using a polydimethylsiloxane micropatterned surface (31). Because *SCN1B^C89/C89^* Pt. 1 and *SCN1B^C89/C89^* Pt. 2 showed similar increases in I_Na_ density, we continued with *SCN1B^C89/C89^* Pt. 1 cells only. Figure 10A shows representative traces of *SCN1B^R89/R89^* control and *SCN1B^C89/C89^* Pt. 1 elicited APs at 1 Hz in current clamp mode. RMP and AP peak amplitude were not significantly different between groups (Figure 10, B and D). In contrast, AP upstroke velocity (dV/dT) for *SCN1B^C89/C89^* Pt. 1 cells was significantly higher compared with *SCN1B^R89/R89^* control, consistent with the observation of increased I_Na_ density in *SCN1B^C89/C89^* Pt. 1 (Figure 10C). In addition, APD was significantly reduced at 20%, 50%, and 90% repolarization, respectively, for *SCN1B^C89/C89^* Pt. 1 compared with *SCN1B^R89/R89^* control (APD90: 453.0 ± 35.3 ms Pt. 1 vs. 667.0 ± 44.3 ms control; Figure 10, E–G), consistent with the observed increased I_to_ and reduced I_Ca,L_ densities recorded in voltage clamp mode.

## Discussion

*SCN1B* variants are linked to DEE52 as well as to cardiac disease, including Brugada Syndrome 5 (BrS5, OMIM 612838) and Atrial Fibrillation Familial 13 (OMIM 615377), although there is evidence to suggest that *SCN1B* may not be a monogenic cause of BrS (32). DEE52 patients have a high rate of SUDEP. We showed previously that *Scn1b*-null mice model DEE52, with spontaneous generalized seizures, ataxia, and a 100% SUDEP rate (19). Because *Scn1b*-null mice also have altered CM excitability resulting in atrial and ventricular arrhythmias (14, 15, 20), we postulated that the high rate of SUDEP in DEE52 may involve cardiac arrhythmias in addition to severe seizures. However, DEE52 patients are not null for *SCN1B*. Thus, we generated a transgenic mouse model of the DEE52 variant *SCN1B-c.265C>T* and demonstrated in previous work that the phenotype of these mice included spontaneous and hyperthermia-induced generalized seizures with a SUDEP rate of 20% (29). In the present study, we investigated the cardiac phenotype of *Scn1b-c.265C>T* mice and then compared our results in mice to iPSC-CMs derived from *SCN1B-c.265C>T* patients to understand the translatability of mouse CM data with regard to DEE52. We show that *Scn1b^C89/C89^* mice, which model biallelic DEE52 patients, have elevated levels of cardiac fibrosis, which can serve as a substrate for the development of cardiac arrhythmias in humans (6). Acutely isolated *Scn1b^C89/C89^* CMs have increased I_to_ density, similar to *Scn1b*-null mice (27). However, AP properties were unchanged likely due to the high variability observed. In contrast, *SCN1B-c.265C>T* DEE52 biallelic patient–derived iPSC-CMs showed increased I_Na_ and I_NaL_ densities, similar to *Scn1b*-null mice (14). Additionally, DEE52 iPSC-CMs had elevated I_to_ density and decreased I_CaL_ densities compared with controls, resulting in AP shortening. Our combined results suggest that, while mouse and human cardiac AP waveforms have critical differences, increased I_to_ density is common to both models of *SCN1B-c.265C>T* DEE52. Taken together, these new results strengthen the hypothesis that cardiac arrhythmias may contribute to SUDEP mechanisms in DEEs linked to variants in VGSC α and β subunit genes.

There is extensive evidence in the literature to show that coexpression of β1 subunits with VGSC α subunits increases I_Na_ in heterologous cells (reviewed in ref. 33). Consistent with these results, acute silencing of *Scn1b* using siRNA knockdown in neonatal rat ventricular CMs dramatically reduced Na_v_1.5 expression and I_Na_ density (34). However, constitutive *Scn1b* deletion in mice has different effects than acute deletion in cells, including aberrant, cell-type-specific changes in the regulation of multiple ionic currents, changes in the regulation of current voltage dependence, and dysregulated gene transcription (14, 15, 19, 20, 27, 35, 36). We attribute this complex phenotype to the combined loss of β1-mediated regulation of ion channel trafficking and voltage dependence as well as loss of β1 RIP–mediated gene modulatory function throughout development. However, because patients are not null for *SCN1B*, we extended our work here by studying mouse and human models of DEE52.

The cardiac phenotype of *Scn1b^C89/C89^* mice recapitulates some, but not all, of our previous findings in *Scn1b*-null mice, suggesting, in agreement with our previous work, that the *SCN1B-c.265C>T* DEE52 variant does not result in complete loss of function (29). *Scn1b^C89/C89^* and *Scn1b*-null mouse CMs have increased functional expression of I_to_ (27) and, similar to other model of epilepsy (37), both mouse strains show increased cardiac fibrosis and high propensity to pacing-induced cardiac arrhythmias (15). In contrast, *Scn1b*-null mouse CMs, but not *Scn1b^C89/C89^* CMs, showed increased I_Na_ and I_NaL_. Both WT β1 and β1-p.R89C polypeptides contain the identical intracellular domain (ICD), which may repress the expression of *Scn5a* mRNA in CMs following translocation of this cleaved fragment to the nucleus. The absence of the β1 ICD in null mice may relieve that repression, resulting in increased *Scn5a* expression. Interestingly, *Scn1b^R89/C89^* CMs showed decreased I_Na_ compared with WT. An intriguing hypothesis to test in future work is that heterophilic cell-cell adhesion between WT and mutant β1 subunits, possibly at the cardiac intercalated disc (25), is deleterious.

In addition to voltage-gated sodium channel α subunits, β1 subunits interact with and modulate the cell surface expression and trafficking of other ion channels, including K_v_4 α subunits (21, 38, 39). Here, the observed increase in *KCND3* mRNA abundance observed in both mouse and human iPSC-derived CM models, with enhanced I_to_ density, support a transcriptional mechanism underlying I_to_ upregulation. While these results indicate a transcriptional contribution, we cannot rule out the additional involvement of posttranscriptional mechanisms, e.g., altered channel trafficking to the plasma membrane, in response to *SCN1B* dysfunction. Interestingly, autonomic dysregulation has been described in *Scn1b*-null mice (14, 15). Because I_to_ is highly sensitive to β-adrenergic stimulation, enhanced adrenergic signaling in *Scn1b-c.265C>T* mice could further augment I_to_ via PKA-mediated phosphorylation of K_v_4 channels or modulation of accessory subunits like KChIP2. Thus, the increased I_to_ observed in our model may reflect a convergence of transcriptional, posttranslational, and autonomic influences, collectively driven by *SCN1B* dysfunction.

While transgenic mouse models have provided valuable insights into potential SUDEP mechanisms, mice are obviously not small humans. Human and mouse ventricular CMs have distinct AP waveforms, due to the differential expression of ion channel genes (40). Thus, to investigate the effects of *SCN1B-c.265C>T* in a human model we generated CMs from patient-derived iPSCs. An additional advantage of iPSC-CM models is that their phenotypes are cell autonomous. Changes in ionic currents in these cells are cell intrinsic rather than the result of remodeling in response to altered autonomic innervation or seizures, as might occur in the whole animal. In our previous work we observed increased I_Na_ density and rates of spontaneous contraction in *SCN1A*-linked DS patient iPSC-CMs (17). For the DS patient with the most markedly increased I_Na_ density, increased incidence of arrhythmogenic AP substrates were recorded from iPSC-CMs, and cardiac and autonomic abnormalities were revealed upon clinical evaluation. Thus, our data were predictive of altered cardiac electrophysiology in 1 individual before cardiac symptoms were diagnosed. We used CRISPR gene editing to generate a heterozygous deletion in *SCN1A* in a non-epileptic control line to ask whether Na_v_1.1 haploinsufficiency alone was sufficient to increase I_Na_ in iPSC-CMs. Similar to the results in DS patient iPSC-CMs, we found an increase in whole-cell I_Na_ density in the CRISPR *SCN1A^+/–^* iPSC-CMs compared with *SCN1A^+/+^* isogenic controls (17). These results suggested that overexpression of another VGSC gene, *SCN5A*, in response to *SCN1A* haploinsufficiency results in altered cardiac excitability in DS.

Here, iPSC-CMs generated from biallelic *SCN1B*-c.265C>T patients showed increased transient and late I_Na_ with an increased rate of AP upstroke. Further electrophysiological characterization demonstrated increased I_to_, reduced I_CaL_, and reduced I_K1_, which together may contribute to the observed AP shortening. Reduced I_K1_ is a hallmark of immaturity, causing iPSC-CMs to fire spontaneously due to depolarized RMP. However, we observed that iPSC-CMs from Pt. 1 did not exhibit spontaneous firing and maintained an RMP below, or less depolarized than, –65 mV, similar to control iPSC-CMs.

Increased I_to_ in both patient-derived iPSC-CMs and *Scn1b^C89/C89^* mouse CMs suggests a conserved electrophysiological phenotype across species. In future work, functional rescue experiments will be required to establish a causal relationship between I_to_ remodeling and the observed electrophysiological phenotypes. Although beyond the scope of the present study, these studies could employ both pharmacological and genetic approaches to modulate or restore I_to_. Pharmacological tools such as 4-aminopyridine (an I_to_ blocker) or NS5806 (an I_to_ enhancer) could be used to assess whether altering I_to_ affects AP waveform properties in CMs or arrhythmia susceptibility and SUDEP in *Scn1b^C89/C89^* mice. The potential contributions of increased adrenergic tone to AP shortening and arrhythmogenesis could be explored using β-adrenergic blockers, which may help distinguish the effects of autonomic imbalance from intrinsic ionic remodeling. Finally, gene replacement strategies targeting K_v_4.3 (*KCND3*) may provide a more selective means to manipulate I_to_ in iPSCs or in mice. These approaches would help delineate whether I_to_ modulation plays a mechanistic role in the pathophysiological features observed in our models and further validate I_to_ as a potential therapeutic target in DEE52.

Clinically, Pt. 1 presented with borderline short PR interval (100 ms), borderline QRS duration (90 ms), and a QTc at the lower end of normal (349 ms). The shorter APD observed in Pt. 1’s iPSC-CMs aligns more closely with the QTc findings in the patient than with our mouse data, which showed a normal QT interval, again supporting the hypothesis that mice cannot fully replicate human cardiac physiology (40). Clinical data from Pt. 1 also suggested right bundle branch block, a conduction abnormality characterized by delayed right ventricular depolarization. Although limited to a single case, this observation suggests potential involvement of the cardiac conduction system in *SCN1B*-related disease. Supporting this hypothesis, *Scn1b^C89/C89^* mice exhibited increased beat-to-beat variability in QRS duration, indicative of ventricular conduction delay. Notably, these mice also displayed increased ventricular fibrosis, which may structurally disrupt conduction pathways such as the right bundle branch. Together, these findings point to a potential mechanistic link between *SCN1B* dysfunction, myocardial fibrosis, and conduction abnormalities, warranting further investigation in both clinical and experimental settings.

In conclusion, our body of DEE patient–derived iPSC-CM work and limited clinical data suggest that the high risk of SUDEP in DS may result from a predisposition to cardiac arrhythmias in addition to neuronal hyperexcitability, reflecting expression of VGSC gene variants in heart and brain.

## Methods

### Sex as a biological variable.

Approximately equal numbers of male and female pups were used in all experiments.

### Animals.

Animals were generated as described previously (29). Animals were housed in the Unit for Laboratory Animal Medicine at the University of Michigan Medical School. Male and female pups were used in all experiments.

### Isolation of mouse ventricular CMs.

Ventricular CMs from P18–P25 mice were isolated using a Langendorff-free method (41). After euthanasia by cervical dislocation, the heart was exposed via sternotomy. Following transection of the descending aorta, HBSS containing 10 mM HEPES, 1 mM MgCl_2_, and 0.5 mM EDTA was immediately flushed into the right ventricle within 1 minute. The heart was then transferred to a 60-mm dish. HBSS without EDTA and supplemented with collagenase type II (Worthington; 280–285 U/mg) was injected into the left ventricle using 10 mL syringe. This procedure was repeated 4 times or until myocytes started to emerge from the heart. Cardiac chambers were separated and gently teared into 1-mm pieces using micro-forceps followed by gentle pipetting. Then the suspension underwent enzymatic activity termination using 10% FBS, passed through a 100-μm strainer, and gravity settling, after which calcium was reintroduced in steps to a final concentration of 1.0 mM. Only quiescent myocytes were used for electrophysiological experiments (41).

### Whole-cell patch clamping recordings.

Patch clamping recordings in isolated mouse CMs and human iPSC-CMs were made using an Axopatch 700B amplifier (Molecular Devices) and pClamp (version 11, Axon Instruments). iPSC-CMs were plated at a low density onto 12-mm glass coverslips coated with 0.1% Matrigel (Corning) and electrophysiological recordings were conducted after approximately 7 days. Patch clamp recordings in iPSC-CMs were conducted between 10 and 60 minutes after replacing the culture medium with the external recording solution and within 4 hours of completing the isolation of mouse CMs. After establishing whole-cell configuration, membrane capacitive components were eliminated, and series resistance was compensated. Additionally, residual non–voltage-dependent currents were eliminated by using a P/4 protocol. This protocol was used for recording of I_Na_ and L-type Ca^2+^ current (I_CaL_). Current traces were normalized against the whole-cell capacitance (C_m_).

Measurements of I_Na_ were obtained using an extracellular solution containing (in mM) 110 CsCl, 1 BaCl_2_, 2 MgCl_2_, 0.2 CdCl_2_, 1 CaCl_2_, 10 HEPES, 20 TEA-Cl, and 10 glucose (pH = 7.35 with CsOH, osmolarity = 300–305 mOsm). This solution was supplemented with NaCl 60 and 10 mM for recording of I_Na_ iPSC-CMs and mouse CMs, respectively. Fire-polished pipettes with resistance of 1.5–2.5 mΩ were filled with internal solution containing (in mM) 1 NaCl, 150 *N*-methyl-D-glucamine, 10 ethyleneglycoltetraacetic acid (EGTA), 2 MgCl_2_, 40 HEPES, and 25 phosphocreatine-tris, 2 MgATP, 0.02 Na_2_GTP, 0.1 leupeptin (pH = 7.2 with H_2_SO_4_). I_Na_ was recorded in response to a series of voltage steps between –120 and +30 mV in 5-mV increments, from a holding potential of –120 mV for 200 ms as described previously (15). A step back to –20 mV for 200 ms was used to determine the voltage dependence of inactivation. Na^+^ conductance (42) at each test voltage was determined from the equation: G_Na_ = I_Na_/(V − E_Na_), where I_Na_ is the sodium current and E_Na_ is the sodium current reversal potential. Peak G_Na_ (G_max_) was plotted as a function of voltage to produce activation curves. I_Na_ was normalized to the maximum elicited current and plotted against the conditioning voltage to yield inactivation curves. Both curves were fitted to the following Boltzmann function: G/G_max_ or I/I_max_=1/(1 + exp[(V − V_½_)/*k*]), where G/G_max_ is the normalized activation and I/I_max_ is normalized inactivation, V_½_ is the voltage of half-maximal activation or inactivation, *k* is the slope factor, and V is the test voltage.

L-type Ca^2+^ current (I_CaL_) was measured using a single pulse to +10 mV from a holding potential of –50 mV, followed by the application of several pulses from –50 to 50 mV in steps of 10 mV to generate an I-V curve. A conditional prepulse to –30 mV was used to inactivate Na^+^ channels. The pipette solution contained (in mM) 120 CsCl-Asp, 10 EGTA-Cs, 1 MgCl_2_, 1 Mg-ATP, 10 TEA-Cl, and 10 HEPES (pH 7.2 with CsOH). The bath solution contained (in mM): 137 NaCl, 5.4 CsCl, 1 MgCl_2_, 1.8 CaCl_2_, 10 HEPES, and 2,4-aminopyridine (pH 7.4 with CsOH). Tetrodotoxin (35 μM; Alomone Labs) was added to the bath solution to ensure the elimination of I_Na_. The steady-state voltage dependence of Na^+^ channel inactivation was assessed using a 2-pulse protocol. This protocol included 200-ms prepulses of variable amplitude, followed by a test pulse to −30 mV. Both the activation and inactivation curves were fitted to the Boltzmann equation, as performed for I_Na_ recordings.

Transient (I_to_) and sustained (I_KSUS_) potassium currents were investigated using repetitive squared 300-ms pulses ranging from –40 to 60 mV. I_K_ collected in the last 50 ms of the recording was defined as I_Ksus_. The difference between I_KSUS_ and the peak of the current collected in the first 50 ms of the recordings was defined as I_to_. Inward I_K_ was recorded from –140 to –30 mV before and after the perfusion of BaCl_2_ (500 μM). I_K1_ was defined as the I_K_ that was sensitive to barium. The pipette solution for I_K_ contained (in mM) 135 KCl, 5 K_2_-ATP, 10 EGTA-K, and 10 HEPES (pH = 7.2 with KOH). The bath solution contained (in mM) 5.3 KCl, 4.1 NaHCO_3_, 138 NaCl, and 100 CaCl_2_ (pH = 7.2 with KOH). CdCl_2_ (250 μM) and nifedipine (10 μM) were added to the bath solution to block calcium currents.

### AP recordings.

iPSC-CMs were recorded on the micron-scale 2-dimensional cardiac muscle bundle (2DMB) platform. To create the 2DMB platform, individual stamps were cut from polydimethoxysiloxane (PDMS), as previously reported (43–45). 2DMB substrates consisted of micropatterned 8 kPa PDMS. Soft PDMS was formulated by mixing Sylgard 527 and Sylgard 184. Each component was first mixed with its own curing agent (i.e., 50:50 for Sylgard 527 and 10:1 for Sylgard 184). In iPSC-CMs and isolated mouse CMs, the threshold for AP initiation was determined by applying 2-ms incremental current pulses ranging from 100 to 1500 pA. Steady-state AP capture was obtained by applying current pulses at 1.5 times the threshold. APs were recorded at 1.0 Hz at room temperature. The bath solution contained (in mM) 135 NaCl, 4 KCl, 1.8 CaCl_2_, 1 MgCl_2_, 10 Hepes, 1.2 NaH_2_PO_4_, 10 glucose, pH 7.35 with NaOH. Patch pipettes were filled with the internal solution containing (in mM) 130 K-aspartate, 10 KCl, 9 NaCl, 0.33 MgCl_2_, 5 Mg-ATP, 0.1 GTP, 10 HEPES, 10 glucose, pH 7.2 with KOH. The RMP was determined under current clamp at zero current.

### RT-qPCR analysis of mouse CMs.

Total RNA was isolated from CM samples using the Qiagen RNeasy Fibrous Tissue Mini Kit according to the manufacturer’s instructions. cDNA was synthesized from 1 μg of total RNA using qScript cDNA Synthesis Kit (QuantaBio, 95047-100). qPCR was performed using SYBR Green (Applied Biosystems) and gene-specific primers (Integrated DNA Technologies) on a QuantStudio 7 Flex Real-Time PCR System (Applied Biosystems). Levels of mRNA abundance were normalized to the internal control, *Gapdh*. The relative abundance levels for each gene were quantified using the comparative threshold (2^−ΔΔCt^) method of quantification.

### Picrosirius red staining.

Heart coronal sections were cut from paraffin-embedded blocks at 5 μm of thickness. Following deparaffinization and hydration with xylene and graded alcohols, the slides were treated with 0.2% phosphomolybdic acid (Rowley Biochemical, F-357-1) for 3 minutes, directly transferred to 0.1% Sirius red saturated in picric acid (Rowley Biochemical, F-357-2) for 90 minutes, then again directly transferred to 0.01N hydrochloric acid for 3 minutes. Slides were dehydrated and cleared through graded alcohols and xylene and coverslipped with Micromount (Leica, 3801731) using a Leica CV5030 automatic coverslipper. Images were acquired with Aperio Digital Pathology Slide Scanners (Aperio GT 450 DX, Leica). The percentage of fibrosis was quantified with ImageJ (NIH) software. Data are presented as mean ± SEM.

### Electrocardiogram and programmed electrical stimulation.

In vivo electrocardiogram (ECG) studies were conducted on P18–P25 anesthetized mice, as described previously (15, 46). Anesthesia was induced with 5.0% (v/v) isoflurane and maintained with 2.0% (v/v) isoflurane in a continuous flow of 100% O_2_ at 0.5 L/min. Once reflexes disappeared, mice were placed on a temperature-regulated operating table. Platinum electrodes were inserted subcutaneously in the limbs and connected to a custom ECG amplifier for standard leads I and II. Standard ECG parameters were analyzed offline, including heart rate (HR), P wave duration, and RR, QRS, PR, and QTc intervals. QTc intervals were analyzed using the Mitchell formula (QTc = QT/√(RR/100). A 1.1 Fr Octapolar stimulation-recording catheter (Scisense, EP catheter) was inserted through the jugular vein and advanced into the right atrium and ventricle. Ventricular and atrial electrical stimulation were performed at twice the threshold of capture. SNRT was measured by delivering 18 pacing stimuli at fixed cycle lengths of 100 ms and 800 ms. AF susceptibility was assessed using trains of 50 electrical pulses at interpulse intervals of 22 ms (45.5 Hz), 20 ms (50 Hz), and 18 ms (55.6 Hz), with each frequency applied 3 times. AF was defined as a rapid, irregular atrial rhythm lacking consistent P waves and characterized by variable atrial cycle lengths with disorganized electrogram morphology lasting at least 1 second. For ventricular arrhythmia assessment, 18 S1 stimuli were delivered at cycle lengths of 100 ms and 80 ms, followed by a single S2 stimulus. The S2 interval was progressively shortened in 2-ms steps, from 40 ms to 16 ms, to determine the ventricular refractory period (VRP), defined as the shortest interval at which ventricular capture failed. This protocol was repeated three times to ensure reliable VRP estimation and to evaluate susceptibility to ventricular tachycardia (VT). Ventricular arrhythmias were defined as either nonsustained ventricular tachycardia (nsVT), characterized by 3 or more consecutive ventricular depolarizations with abnormal QRS morphology distinguishable from baseline rhythm, or ventricular fibrillation (VF), defined as rapid, disorganized electrical activity lacking discernible QRS complexes and lasting at least 1 second. Both nsVT and VF episodes were considered indicative of inducible ventricular arrhythmias.

### Human iPSCs.

iPSCs were reprogrammed by the episomal plasmid method with Neon Transfection system (Life Technologies). *SCN1B^R89/R89^* control 1 (Ctrl1) and *SCN1B* patient 1 (Pt1-8 and Pt1-10; referred to here as Pt. 1 and Pt. 2) iPSC lines were generated from skin fibroblast biopsies as previously described (17). *SCN1B^R89/C89^* control (Het control) and Pt. 2 iPSC lines were generated from PBMCs by commercially available methods (Stem Cell Genetics). *SCN1B^R89/R89^* control 2 (Ctrl2-2) were obtained from the Human Stem Cell and Gene Editing Core at the University of Michigan. iPSCs were maintained in feeder-free conditions on 0.5% Matrigel–coated plates (Corning) in mTeSR1 medium (Stem Cell Technologies), passaged every 4–5 days with 0.1 mM EDTA as described previously (17). Medium was changed daily. Genomic alteration of iPSCs were checked by SNP-CHIP at cell passages 10–20. The cells were cultured in 37°C with 5% CO_2_.

### iPSC-CM differentiation.

iPSCs were differentiated to CMs using a small molecule patterning method, based on Wnt signaling pathway modulation (30). Briefly, iPSCs were dissociated by Accutase (STEMCELL Technologies) and plated at 1 × 10^6^ to 1.5 × 10^6^ cells/well in mTeSR-1 on 6-well plates coated with 1% Matrigel. When the cells reached greater than 90% confluence, differentiation was initiated. The cells were cultured in a basal medium (RPMI/B27 without insulin [Thermo Fisher Scientific]) for approximately 10 days. During the first 24 hours of differentiation, the basal medium contained 6 mM glycogen synthase kinase-3β inhibitor CHIR99021 (Cayman Chemical). On days 3–5 of differentiation, the basal medium included 5 mM Wnt inhibitor IWP4 (Stemgent). On days 10–14, the basal medium was changed, and the cells were cultivated in maintenance medium (RPMI/B27 with insulin). Between days 8 and 14 of differentiation, the cells began to spontaneously contract. On day 14 of differentiation, cells were maintained in a medium containing lactate enrichment medium (RPMI, no glucose with HEPES, bovine serum albumin, lactate, and L-ascorbic acid (all Thermo Fisher Scientific]) (47) for 4–8 days. The cells were then changed to maintenance medium, which was changed every 2–3 days until day 40. The cells were dissociated by TrypLE (Thermo Fisher Scientific) and 0.5 × 10^4^ to 1.2 × 10^4^ cells were plated on Matrigel-coated 12-mm^2^ glass coverslips in maintenance medium for electrophysiological analyses, immunostainings, and image analyses.

### RT-qPCR analysis in iPSC-CMs.

Total RNA was isolated from samples using the Qiagen RNeasy Fibrous Tissue Mini Kit according to the manufacturer’s instructions. cDNA was synthesized from 1 μg of total RNA using qScript cDNA Synthesis Kit (QuantaBio, 95047-100). qPCR was performed using SYBR Green (Applied Biosystems) and gene-specific primers (Integrated DNA Technologies) on a QuantStudio 7 Flex Real-Time PCR System (Applied Biosystems). The mRNA level was normalized to the internal control, GAPDH. The relative expression levels for each gene were quantified using the comparative threshold (2^−ΔΔCt^) method of quantification.

### Statistics.

Data are presented as the fold change in gene expression ± SEM. Statistical significance of comparisons between genotypes was determined using a Student’s *t* test for comparisons between 2 variables, or a 1-way ANOVA with Tukey’s post hoc comparison test for comparisons involving more than 2 variables. A *P* value of less than 0.05 was considered significant.

### Study approval.

All animal procedures were performed in accordance with NIH policy and approved by the University of Michigan Institutional Animal Care and Use Committee (PRO00010562). The human iPSC work was performed under approval from the University of Michigan Human Plurioptent Stem Cell Research Oversight Committee.

### Data availability.

The datasets used and/or analyzed during the current study are available from the corresponding author on reasonable request. Values for all data points in graphs are reported in the Supporting Data Values file.

## Author contributions

RRM conducted patch clamp electrophysiological recordings in isolated mouse CMs and hiPSC-CMs and performed mouse intracardiac recording. SW performed cardiac myocyte isolation, Picrosirius red staining, imaging, qPCR, and data analysis. NE performed patch clamping experiments in hiPSC-CMs. QL assisted with patch clamping recordings in mouse CMs. MS contributed with the patch clamping data analysis. XQ assisted with the culture and maintenance of hIPSC-CMs. LFLS analyzed electrocardiogram data. ASH and SW provided patient samples. LTD, YCT, and KS contributed with the design of the protocols for the development of hIPSC-CMs. LLI and JMP contributed to experimental design and interpretation and provided funding. RRM, NE, and LLI co-wrote the manuscript.

## Supplementary Material

Supplemental data

Supporting data values

## Version 1 08/05/2025

In-Press Preview

## Version 2 09/09/2025

Electronic publication
