# Supplementary material for: Altered cardiac excitability and arrhythmia in models of SCN1B-linked developmental and epileptic encephalopathy
Source: JCI Insight. 2025 Aug 5;10(17):e190918. doi: 10.1172/jci.insight.190918 (PMC12487680; doi:10.1172/jci.insight.190918)
Supplement: Supplemental data [file jciinsight-10-190918-s276.pdf]

SUPPLEMENTAL MATERIALS

Altered Cardiac Excitability and Arrhythmia in Models of *SCN1B*-Linked Developmental and Epileptic Encephalopathy

Roberto Ramos-Mondragon[1], Shuyun Wang[1], Nnamdi Edokobi[1], Qinghua Liu[1], Xiaotan Qiao[2], Maya Shih[3], Louis T. Dang[4], Yao-Chang Tsan[5], Katalin Štěrbová[6], Adam S. Helms[6], Sarah Weckhuysen[7,8,9], Luis Lopez-Santiago[1], Jack M. Parent[2,10,11], Lori L. Isom*[1,2]

Author affiliations:

Departments of Pharmacology[1], Neurology[2], Neuroscience[3], Pediatrics[4], Human Genetics[5], Internal Medicine[6], University of Michigan Medical School, Ann Arbor, MI 48109

[6]Department of Pediatric Neurology, Charles University and Motol Hospital, Prague, Czech Republic

[7]Applied&Translational Neurogenomics Group, VIB Center for Molecular Neurology, VIB, Antwerp, Belgium

[8]Translational Neurosciences, Faculty of Medicine and Health Science, University of Antwerp, Antwerp, Belgium

[9]Department of Neurology, Antwerp University Hospital, Antwerp, Belgium

[10]Michigan Neuroscience Institute, University of Michigan Medical School, Ann Arbor, MI 48109

[11]VA Ann Arbor Healthcare System, Ann Arbor, MI 48105

Correspondence to: Lori L. Isom, Ph.D., Department of Pharmacology, University of Michigan Medical School, Ann Arbor, MI 48109, lisom@umich.edu, 734-936-3050

Supplemental Figures and Figure Legends

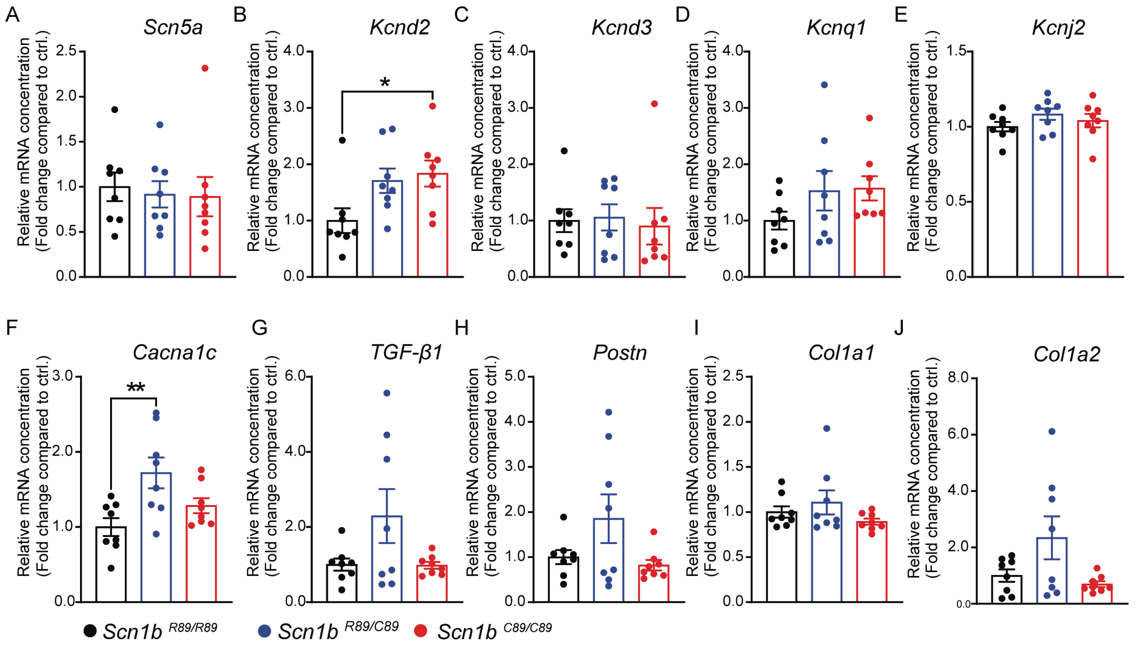

Figure S1. mRNA abundance of ion channel and extracellular matrix regulatory genes in *Scn1b*$^{R89/R89}$, *Scn1b*$^{R89/C89}$ and *Scn1b*$^{C89/C89}$ mice determined by RT-qPCR. mRNA abundance of (A) *Scn5a*, encoding for Na$_v$1.5 channel, (B) *Kcnd2*, encoding K$_v$4.2 channel, (C) *Kcnd2*, encoding for K$_v$4.3 channel , (D) *Kcnd2*, encoding for K$_v$7.1 channel, (E) *Kcnj2*, encoding Kir2.1 channel, (F) *Cacna1c*, encoding for Ca$_v$1.2 channel, (G) *Tgfb1*, encoding for TGF-β1, (H) *Postn*, encoding for periostin, (I) *Col1a1*, encoding for collagen type 1, and (J) *Col1a2*, encoding for collagen type 2. Data are represented as mean ± SEM. Each dot represents and individual measurement. *$*p<0.05$* and *$*p<0.01$* using a one-way ANOVA with Tukey's post-hoc comparison test.

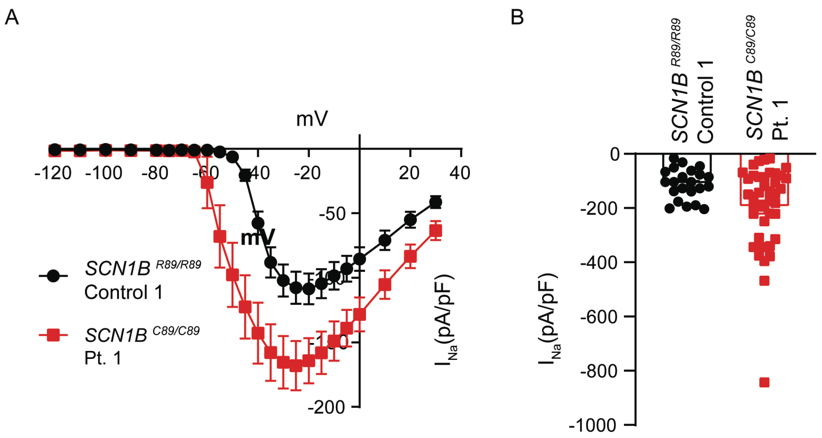

Figure S2. I_{Na} density in patient 1 iPSC-CMs. (A) I_{Na} current-voltage relationship for *SCN1B*$^{R89/R89}$ control and Pt. 1 iPSC-CM lines using an external recording external solution containing 120 mM NaCl. (B) I_{Na} density is increased in Pt. 1 vs. *SCN1B*$^{R89/R89}$ control iPSC-CMs, however, consistent voltage control could not be maintained in Pt. 1 iPSC-CMs. Data are represented as mean ± SEM. n= 22 cells from *SCN1B*$^{R89/R89}$ control 1 and n=42 cells from *SCN1B*$^{C89/C89}$ Pt. 1. All cells were derived from at least 3 independent hiPSC differentiation batches. Dots represent individual cells.

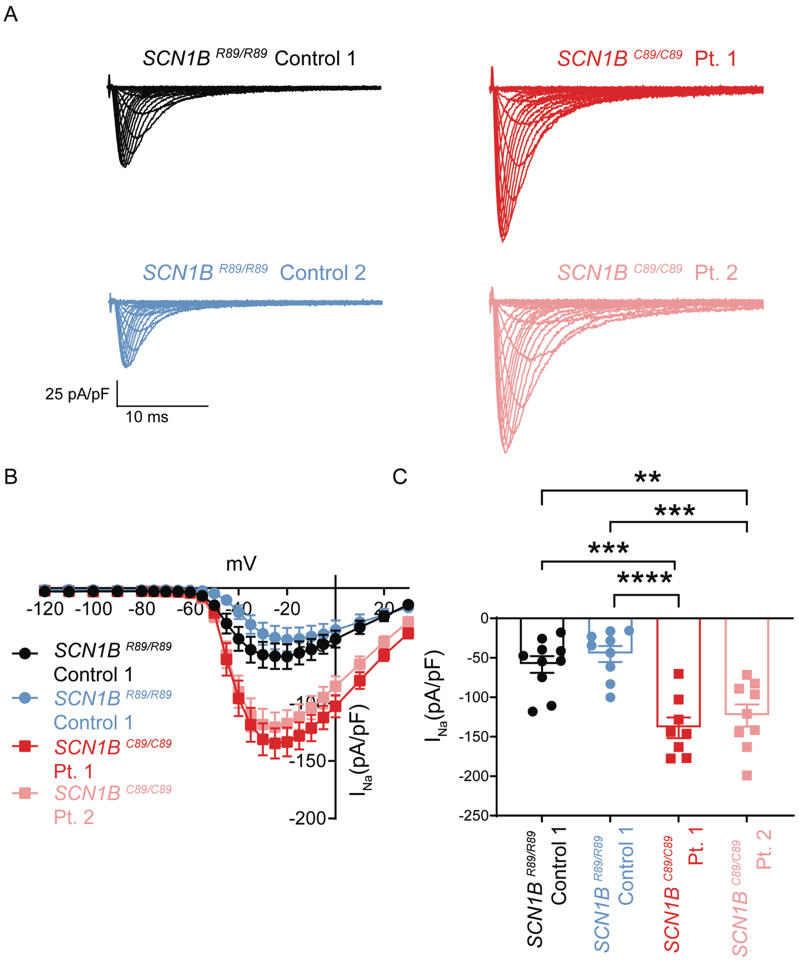

Figure S3. Independent representations of pooled data. (A) Representative I_{Na} density traces. (B) I_{Na} current-voltage relationship for *SCN1B*$^{R89/R89}$ control 1, *SCN1B*$^{R89/R89}$ control 2, and Pt. 1 iPSC-CM clonal lines. (C) Transient peak I_{Na} density is increased 2-fold in Pt. 1 clones vs controls. Data in panel C are presented as means ± SEM. n= 9 cells from *SCN1B*$^{R89/R89}$ control 1, n= 9 cells from *SCN1B*$^{R89/R89}$ control 2, n=8 cells from *SCN1B*$^{C89/C89}$ Pt. 1, n=10 cells from *SCN1B*$^{C89/C89}$ Pt. 2. All cells were derived from at least 3 independent hiPSC differentiation batches *$p<0.05$, **$p<0.01$, ***$p<0.001$ and ****$p<0.0001$ using one-way ANOVA with Tukey's post-hoc comparison test. Dots represent individual cells.

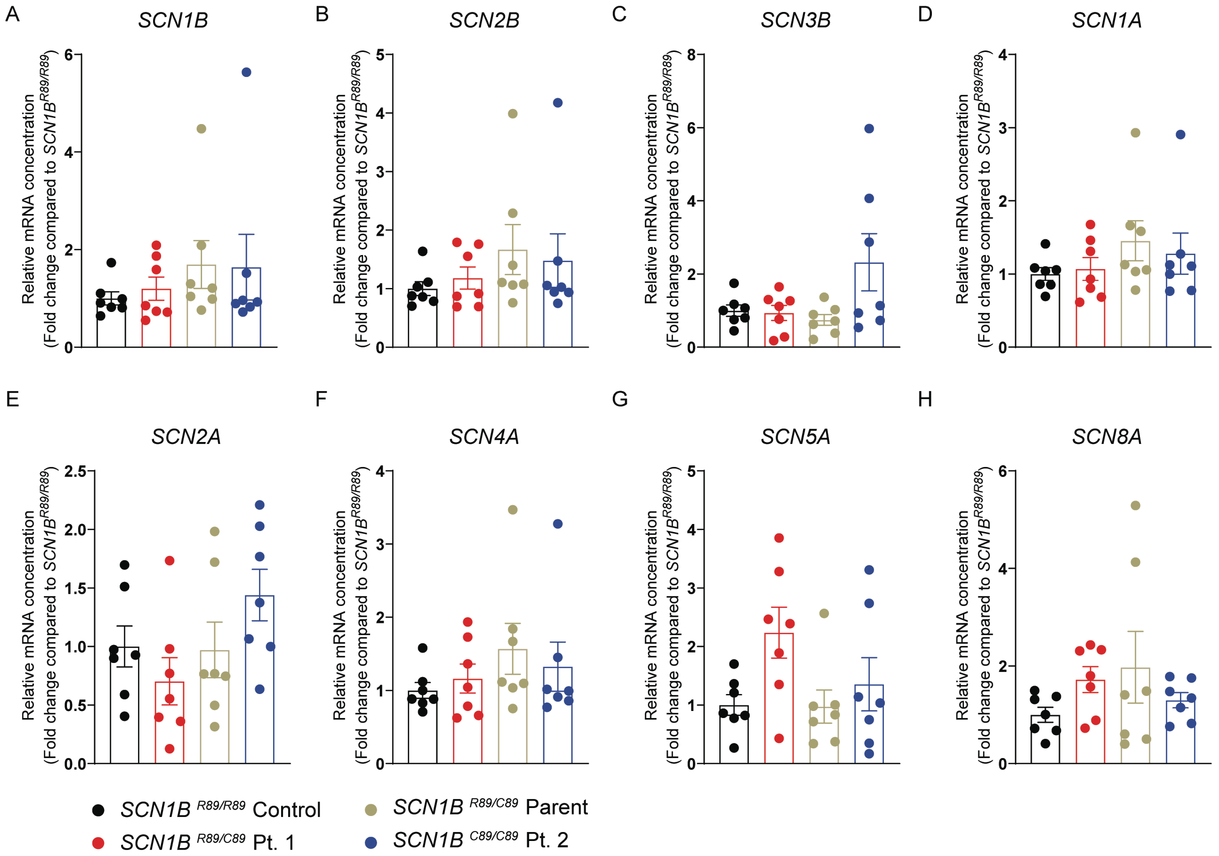

Figure S4. VGSC α and β subunit mRNA abundance in patient-derived iPSC-CMs determined by RT-qPCR. Quantification of the mRNA levels of (A) *SCN1B*, encoding for β1 sodium channel subunit, (B) *SCN2B*, encoding for β2 sodium channel subunit, (C) *SCN3B*, encoding for β3 sodium channel subunit, (D) *SCN1A*, encoding for α-subunit of $Na_v1.1$ channel, (E) *SCN2A*, encoding for α-subunit of $Na_v1.2$ channel, (F) *SCN4A*, encoding for α-subunit of $Na_v1.4$ channel, (G) *SCN5A*, encoding for α-subunit of $Na_v1.5$ channel and (H) *SCN8A*, encoding for α-subunit of $Na_v1.6$ channel in iPSC-CMs derived from *SCN1B*$^{R89/R89}$ control, *SCN1B*$^{C89/C89}$ Pt. 1, *SCN1B*$^{R89/C89}$ parent and *SCN1B*$^{C89/C89}$ Pt. 2. Data are represented as the mean ± SEM. Each dot represents and individual measurement. Statistical differences were assessed by one-way ANOVA with Tukey's post-hoc comparison test.

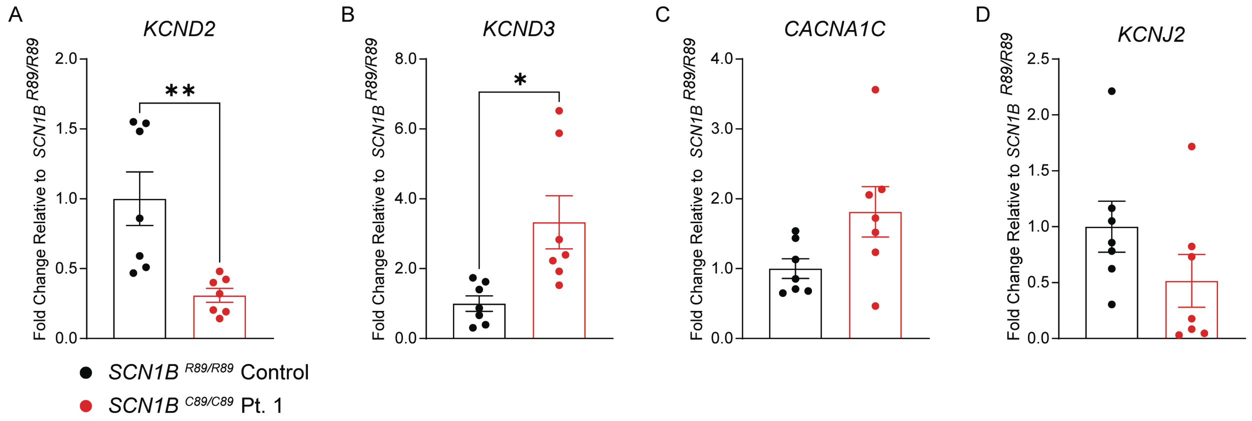

Figure S5. Potassium and L-type Ca^{2+} channel mRNA abundance determined by RT-qPCR. Quantification of the mRNA levels of (A) *KCND2* encoding for K_v4.2 channel, (B) *KCND3* encoding for Kv4.3 channel, (C) *CACNA1C* encoding for Ca_v1.2 C channel and (D) *KCNJ2* encoding for Kir2.1 channel in *SCN1B*$^{R89/R89}$ control 1 and *SCN1B*$^{C89/C89}$ Pt. 1 derived iPSC-CMs. *SCN1B*$^{C89/C89}$ iPSC-CMs showed decreased *KCND2* and increased *KCND3* expression compared to *SCN1B*$^{R89/R89}$ iPSC-CMs. Data are represented as the mean ± SEM. Each dot represents an individual measurement. *$p<0.05$ and ** $p<0.01$ using unpaired two-tailed Student's t-test.

Supplemental Tables

	Scn1b$^{R89/R89}$	*Scn1b*$^{R89/C89}$	*Scn1b*$^{C89/C89}$
Body weight (g):	9.4 ± 0.5	9.5 ± 0.5	9.3 ± 0.6
Heart weight (mg):	97.0 ± 5.0	92.0 ± 5.4	93.1 ± 6.2
HW/BW (mg/g):	10.0 ± 0.2	10.0 ± 0.5	10.0 ± 0.4
**ECG-I**			
HR (bpm)	470.7±17.3	455.8±13.8*	446.7±15.0
P wave (ms)	11.9±0.7	11.9±0.5	12.2±0.7
PR-I (ms)	37.0±1.1	36.80±0.9	37.8±1.5
QRS-I (ms)	9.8±0.3	10.0±0.3	10.8±0.7
QT-I (ms)	51.9±4.1	46.3±2.7	53.5±3.0
QTc-I (ms)	48.0±4.8	39.9±2.1	45.8±2.4
**ECG-II**			
HR (bpm)	470.7±17.3	455.8±13.8*	445.8±15.4
P wave (ms)	16.0±0.9	16.1±1.0	15.4±1.3
PR-I (ms)	36.1±4.0	36.7±1.1	37.0±1.4
QRS-I (ms)	11.6±0.5	11.7±0.4	12.9±1.2
QT-I (ms)	57.3±2.6	58.6±1.4	59.7±3.7
QTc-I (ms)	52.5±3.4	50.7±0.8	50.9±2.5
N	16	30	16

Supplemental Table 1. Heart weight to body weight relationship and ECG parameters in P18-25 anesthetized transgenic mice. QT was corrected using Mitchell's formula. Data are presented as mean ± SEM. *$p<0.05$ against *SCN1B*$^{R89/R89}$ using a One-Way Anova with Tukey's post-hoc comparison test.

	Scn1b$^{R89/R89}$ CMs	*Scn1b*$^{R89/C89}$ CMs	*Scn1b*$^{C89/C89}$ CMs
**Voltage-dependent activation**			
G_{max} (mV)	81.0 ± 5.6	60.7 ± 4.4*	81.0 ± 7.6
$V_{1/2}$ (mV)	-41.7 ± 1.0	-43.6 ± 1.2	-41.6 ± 0.8
k (mV)	3.5 ± 0.3	3.5 ± 0.3	3.6 ± 0.2
V_{rev} (mV)	37.1 ± 2.2	48.2 ± 6.2	35.0 ± 2.0
**Voltage-dependent inactivation**			
I_{max} (pA)	4733.4.0 ± 388.7	4118.3 ± 293.0	4454.7 ± 286.3
$V_{1/2}$ (mV)	-8.2 ± 0.2	-8.8 ± 0.2	-7.6 ± 0.3$^{\#}$
Cm (1)	76.5 ± 4.1	95 ± 6.2*	71.4 ± 3.5
n	19	24	23

Supplemental Table 2. Voltage dependent activation and inactivation for I_{Na} recorded in acutely isolated P18-25 *Scn1b*$^{R89/R89}$, *Scn1b*$^{R89/C89}$, and *Scn1b*$^{R89/C89}$ ventricular mouse CMs. Data are presented as mean ± SEM. *$p<0.05$* against *Scn1b*$^{R89/R89}$ and *Scn1b*$^{R89/C89}$ and # *$p<0.05$* against *Scn1b*$^{R89/C89}$ by using One-Way Anova with Tukey's post-hoc comparison test.

	Scn1b$^{R89/R89}$ CMs	*Scn1b*$^{R89/C89}$ CMs	*Scn1b*$^{C89/C89}$ CMs
**Voltage-dependent activation**			
G_{max} (nS)	18.0 ± 1.5	18.5 ± 1.5	20.4 ± 1.7
$V_{1/2}$ (mV)	-3.9 ± 0.4	-3.9 ± 0.5	-5.1 ± 0.7
k (mV)	7.4 ± 0.2	7.3 ± 0.3	7.1 ± 0.2
V_{rev} (mV)	59.7 ± 0.4	61.2 ± 0.5	61.2 ± 0.6
**Voltage-dependent inactivation**			
I_{max} (pA)	608.3 ± 66.7	795.0 ± 86.5	836.8 ± 111.0
$V_{1/2}$ (mV)	-11.7 ± 1.8	-8.9 ± 1.0	-8.0 ± 0.3
C_m (1)	81.5 ± 4.7	84.2 ± 3.8	85.0 ± 4.1
n	30	23	25

Supplemental Table 3. Voltage-dependent activation and inactivation for I_{CaL} recorded in P18-25 acutely isolated *Scn1b*$^{R89/R89}$, *Scn1b*$^{R89/C89}$, and *Scn1b*$^{R89/C89}$ ventricular mouse CMs. Data are presented as mean ± SEM. No significant differences were found between groups when using a One-Way Anova with Tukey's post-hoc comparison test.

	Scn1b$^{R89/R89}$	*Scn1b*$^{R89/C89}$	*Scn1b*$^{C89/C89}$
$SNRT_{100}$ (ms)	158.1 ± 13.3	176.5 ± 13.0	147.4 ± 12.0
$SNRT_{80}$ (ms)	181.4 ± 17.7	201.0 ± 17.3	159.0 ± 14.5
$VERP_{100}$ (ms)	20.0 ± 1.3	21.2 ± 1.2	18.0 ± 1.3
$VERP_{80}$ (ms)	21.5 ± 1.0	22.3 ± 1.3	18.0 ± 1.5*
VT duration (s)	0.23 ± 0.0	0.26 ± 0.0	0.28 ± 0.0$^{\#}$
AF duration (s)	2.0 ± 0.2	4.1 ± 0.5	3.9 ± 1.0
N=	8	8	6

Supplemental Table 4. Programmed electrical stimulation parameters in P18-25 mice. Data are presented as mean ± SEM. *$p<0.05$* against *SCN1B*$^{R89/C89}$ and $^{\#}$*$p<0.05$* against *SCN1B*$^{R89/R89}$ using a One-Way Anova with Tukey's post-hoc comparison test.

	**Patient 1 Clone 1**	**Patient 1 Clone 2**	**Patient 2**	**Control 1**	**Control 2**	**Mother of Patient 2**
**Genotype**	*SCN1B*$^{C89/C89}$	*SCN1B*$^{C89/C89}$	*SCN1B*$^{C89/C89}$	*SCN1B*$^{R89/R89}$	*SCN1B*$^{R89/R89}$	*SCN1B*$^{R89/C89}$
**Sex**	M	M	F	F	M	F
**Biopsy**	dermal fibroblasts	dermal fibroblasts	PBMCs	dermal fibroblasts	dermal fibroblasts	PBMCs

Supplemental Table 5. Information on iPSC-CM patient lines.

	SCN1B$^{R89/R89}$ control 1 and 2, pooled data	*SCN1B*$^{R89/C89}$ parent	Patient 1	Patient 2
**Voltage dependence of activation**				
$V_{1/2}$ (mV)	-36.59 ± 0.43	-38.66 ± 0.41	-39.92 ± 0.29	-41.57 ± 0.37
k (mV)	6.87 ± 0.38	6.33 ± 0.6	5.90 ± 0.25	6.28 ± 0.33
G_{max} ($_{pS}$)	16.8 ± 2.5	13.8 ± 2.7	40.0 ± 4.6*	38.1 ± 7.3*
n	19	13	17	16
**Voltage dependence of inactivation**				
$V_{1/2}$ (mV)	-72.83 ± 0.66	-70.89 ± 0.61	-73.64 ± 0.41	-69.64 ± 0.32
h (mV)	-8.78 ± 0.58	-8.10 ± 0.54	-8.81 ± 0.36	-7.65 ± 0.28
n	19	13	17	16
Capacitance (1)	18.54 ± 2.58	19.70 ± 1.67	23.77 ± 2.90	21.89 ± 2.25

Supplemental Table 6. I_{Na} properties of iPSC-CMs. Data are presented as mean ± SEM. *$p < 0.001$ versus Control and Het control using a one-way ANOVA with Tukey's post-hoc comparison test.
